# Anthocyanins in Colorectal Cancer Prevention. A Systematic Review of the Literature in Search of Molecular Oncotargets

**DOI:** 10.3389/fphar.2019.00675

**Published:** 2019-06-21

**Authors:** Nevenka Medic, Federica Tramer, Sabina Passamonti

**Affiliations:** Department of Life Sciences, University of Trieste, Trieste, Italy

**Keywords:** anthocyanins, colorectal cancer, oncotargets, systematic review, diet

## Abstract

**Background:** Colorectal cancer (CRC) is the malignant process that surges in the terminal part of gastrointestinal tract when adenomatous polyps convert to neoplastic cells able to infiltrate the submucosa. Despite the constant progress in applying preventive measures (screening, colonoscopy) and developing new cures (surgical and chemotherapy), CRC is still one of the leading causes of cancer death worldwide. The importance of natural dietary components in CRC prevention has been recognized. Defining the precise role of the diet and its particular molecular moieties in CRC prevention is of constant scientific interest years behind. Anthocyanins (AC), phenolic phytochemicals present in pigmented plants and vegetables, have been reported to have some role in counteracting CRC carcinogenesis. Nonetheless, evidence coming out the pre-clinical, clinical, and epidemiological studies is still controversial. This review is addressing the need to better comprehend the causes of missing data and discrepancies in investigations on the role of dietary AC in modulating CRC carcinogenesis.

**Methods:** We have analyzed the scientific literature, available in PubMed database, according to PRISMA (Preferred Reporting Items for Systematic Reviews and Meta-Analyses) statement methodology for systematic reviews. Subsequently, two selection strategies, with their screening and eligibility criteria, were applied to retain research articles reporting *in vitro* and *in vivo* studies aimed at exploring the molecular mechanisms underlying the observed effects of AC in CRC prevention.

**Results:** From the pool of 82 identified publications, we selected 19 articles reporting experimental or observational data on the effect of AC-enriched diets in CRC prevention in humans or murine species. Furthermore, we selected 10 articles reporting about molecular mechanisms of action of pure AC in CRC experimental models.

**Conclusions:** The major outcome of this review is that AC showed essentially no effect in human studies, whereas AC-enriched diets proved to be effective in experimental murine models of CRC. In cell culture tests, AC showed to interfere with cell signaling pathways related to cell growth and differentiation, apoptosis, oxygen stress, and inflammation response. Further molecular characterizations are required to include AC in the panel of disease-modifying agents.

## Introduction

Colorectal cancer (CRC) is the third most common cancer and the fourth most common cause of cancer death worldwide. Even though there is a remarkable advancement in the field of early detection throughout programmed screenings as well as therapies like surgery and chemotherapy, there were still over 1.8 million new cases in 2018 (Bray et al., 2018).

Cancer arises in the terminal part of the intestine, triggered by mutations in oncogenes or tumor suppressor genes expressed in crypt cells. Cumulative mutations lead to uncontrolled division of mucosal cells and formation of polyps. Some of these transform to precancerous adenomas and subsequently to neoplasia. Early detectable change in mucosa is characterized by the presence of the aberrant crypt foci (ACF) (Takayama et al., 1998; Janakiram and Rao, 2008).

Risk factors are related to age, sex, or family history. What is interesting, CRC has a rising incidence connected to the changeable risk factors, i.e., those related to lifestyles and environmental exposure, like physical inactivity, diets rich in processed and red meats, smoking, and alcohol consuming. CRC is also correlated with economic and consequent nutritional transition, and it is increasing in countries that are undergoing rapid social and economic changes (Bray et al., 2012).

The scientific community has perceived these evidences and focused its attention on studies on the relation of food consumption with CRC prevention. A large portfolio of evidence associating high intake of vegetables, fruits, whole grains, and foods containing dietary fiber, dairy products, and calcium supplements with the declined risk of CRC is available (Ryan-Harshman and Aldoori, 2007). It has been hypothesized that specific phytochemicals might be the chemical agents of CRC prevention, raising the hope that these molecules may act synergistically with conventional chemotherapeutics (Shin et al., 2009).

Anthocyanins (AC) are natural compounds that have attracted attention in the recent years for their capacity to interact with cancer cells and modify their cellular response. Due to their phenolic structure and the presence of hydroxyl groups, AC are also recognized as a potent antioxidants. By quenching reactive oxygen species, AC prevent mutagenesis in normal cells, but may be toxic for cancer cells (Wang and Stoner, 2008; Cvorovic et al., 2010).

### Objectives of the Study

We were interested in performing a systematic analysis of the literature to find information from preclinical, clinical, and epidemiological studies on the mechanism by which AC prevent the onset and progression of CRC, and the underlying molecular events. Thus, we read the scientific literature to seek information and address a series of specific questions, such as the following:

1. Are there any studies performed on human groups or populations? The aim was to provide an overview of the available knowledge about the ability of AC-containing food to prevent human colorectal cancer.
2. Are there any studies performed on animals? What animal models have been used? The aim was to survey investigations on the effects of AC-rich foods on experimental CRC in animals.
3. Are there any studies performed in cellular models? The aim was to survey *in vitro* studies performed in cellular models of CRC, focusing on the effect(s) of pure AC on the molecular process of carcinogenesis.
4. What molecules have been used to test anti-cancer effects? The aim was to check what AC molecules were tested.
5. Which biological processes have been investigated? The aim was to survey the mechanisms whereby AC interfere with usual carcinogenic pathways.
6. Have specific molecular targets or markers been identified? The aim was to survey AC molecules that may potentially serve as anticancer drugs.

It is easily recognizable that this series of questions recapitulates the approach of reverse translational medicine to drug discovery, which is also said “bedside-to-bench” (Gibbs et al., 2018). By this approach, observations about patients and their response to treatments are the starting point to design and simultaneously implement more detailed pharmacokinetic and pharmacodynamic tests, as well as more targeted, hypothesis-driven *in vitro* molecular studies. This approach is believed to accelerate drug development, since it focuses on a limited number of drug candidates. Furthermore, all phases of drug discovery can be run at the same time.

To search for answers to these questions, we have applied the PRISMA Statement methodology (Shamseer et al., 2015) to retrieve, select, and analyze the scientific bibliography about the role of AC in CRC by a standardized method, which can be easily replicated and will enable continuous updating of the research outcomes.

## Materials and Methods

### Identification of Articles in Public Database

#### Information Sources

We have searched PubMed, regarded as the most important and complete archive of biomedical literature.

#### Search Strategy

We have searched for documents in PubMed database by using the query “colorectal cancer AND anthocyanins.” **Table 1** summarizes the search strategy details. Filters limits were not applied. The time window was up to 31.12.2018.

**Table 1:** Search strategy: details of database query.

Key word	MeSH ID	MeSH heading	Rationale
Colorectal cancer	D015179	Tumors or cancers of the colon or the rectum or both	This systematic review is focused on this particular type of neoplasm. AC are poorly absorbed. A large fraction transits in the intestine and reaches the colon (Williamson et al., 2018), which is the organ that is exposed to the highest concentrations of AC and their catabolites.
Anthocyanins	D000872	A group of FLAVONOIDS derived from FLAVONOLS, which lack the ketone oxygen at the 4-position	This systematic review is focused on the effects of only AC in CRC prevention. The average intake of dietary AC is the highest among the various sub-classes of flavonoids (Zamora-Ros et al., 2011).

AC, anthocyanins; CRC, colorectal cancer.

### Selection of Eligible Articles

#### Selection Strategy

The articles identified during the search step were first screened to retain only research articles complying with criteria specified in **Table 2**.

**Table 2:** Pre-selection.

PHASE	TEXT	IN	OUT	RESULTS
First level screening	Title (Abstract, if needed)	Research articles In English	Not in English Reviews Letters to the editor Commentaries Other tumor types (not CRC) Other factors, indirectly related to CRC	Articles screened for further eligibility check

The retained documents were analyzed by applying two parallel selection strategies. Strategy A (**Table 3**) was applied to identify studies showing if AC-rich food reduces the risk of CRC. Strategy B (**Table 4**) was applied to identify studies highlighting the molecular mechanisms by which pure AC compounds, either alone or in pure mixtures, interact with hypothetical molecular targets involved in colorectal carcinogenesis. In respect to the experimental use of pure AC mixtures, it was decided that the purity threshold for inclusion was 90% (w/w).

**Table 3:** Selection strategy A: role of AC-enriched diet in the CRC prevention.

PHASE	TEXT	IN	OUT	RESULTS
Second level screening	Abstracts (with Materials and Methods, if needed)	Food or extracts containing AC Human or murine studies Epidemiological or interventional studies	Not human or murine studies *In vitro* studies Studies using AC metabolites	Articles screened for further eligibility check
Eligibility check	Full text	“Materials and Methods” section describes: a) clear dietary plan (or AC intake is estimated by survey); b) relative risk (RR) calculated “Results” section (including Figures and Tables) reports statistical analyses on AC protective effects	Statistical analyses not reported	Included articles as eligible for the analysis of their study features

**Table 4:** Selection strategy B: AC-related anti-cancer molecular mechanisms.

FASE	TEXT	IN	OUT	RESULTS
Second level screening	Abstracts (with Materials and Methods, if needed)	Studies where either pure AC or >90% AC extracts have been used Mammalian experimental models (mice, rats, human cell lines)	AC extracts with purity < 90% Studies using AC metabolites Studies on nonmammalian models Studies not reporting anticancer effects by a definite biological interaction QSAR studies	Articles screened for further eligibility check
Eligibility check	Full text	“Materials and Methods” section describes at least one bioassay to assess anticancer effect(s) with definite biological interaction (AC-protein, AC-DNA, AC-RNA, AC-polysaccharide) The article section “Results” (including Figure and Tables) reports experimental data about anticancer activity with definite biologic interaction	Anticancer biological activity was only hypothesized	Included articles as eligible for the analysis of their study features

Eligible articles were retained for full-text reading, analysis, and classification of their study features are expected to provide answers on the abovementioned research questions.

**Table 5** presents a synopsis of the rationale applied in this systematic review. Indeed, for each main question, a specific selection strategy (i.e., either A or B) was applied to select articles from the pool.

**Table 5:** Rationale behind this systematic review. Each research question was addressed by a given selection strategy (either A or B). Included articles were read to identify a number of study features.

Research questions	Selection strategy	Study features examined in included articles
1) Are there any studies performed on human groups or populations?	Role of AC-enriched diet in CRC prevention	Human population/group of people subjects n° Food or extracts Dose of AC Type of study Study duration Risk reduction
2) Are there any studies performed on animals? What animal models have been used?	Role of AC-enriched diet in CRC prevention	Animal model, subjects n° Food or extracts Dose of AC Type of study Study duration Risk reduction
3) Are there any studies performed in cellular models?	AC-related Anti-cancer Molecular Mechanisms	Cells
4) What molecules have been used to test their anti-cancer effect?	AC-related Anti-cancer Molecular Mechanisms	AC molecules or mixtures of thereof
5) Which biological processes have been investigated?	AC-related Anti-cancer Molecular Mechanisms	Reaction, pathway or function modified by AC
6) Have specific molecular targets or markers been identified?	AC-related Anti-cancer Molecular Mechanisms	Molecular target Molecular marker

## Results

### Literature Search and Selection

**Figure 1:**
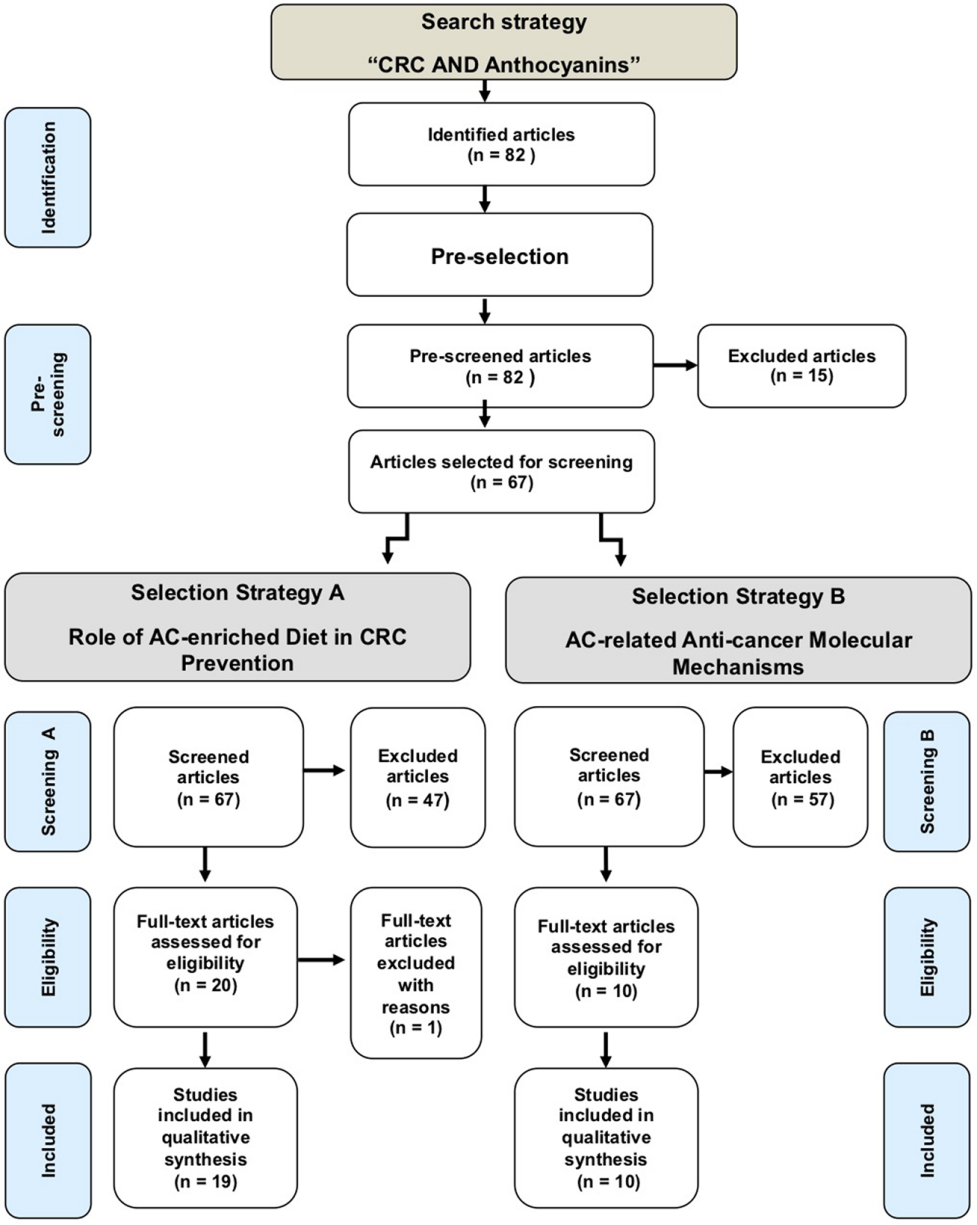
Flowchart of the output of the literature search strategy and screenings.

**Figure 1** is the flowchart of this systematic review. It shows that 82 articles were identified in PubMed. After pre-screening, 67 articles were screened for inclusion. The output was 19 and 10 studies, resulting from strategies A and B, respectively (**Supplementary Material**).

### Role of AC-Enriched Diet in the CRC Prevention

Nineteen studies were analyzed for features, deemed important for gaining an insight in the role of AC in CRC prevention. **Table 6** provides a synopsis of these features. Concerning the species on which AC diets have been tested, studies reported data collected in humans (*n* = 4), mice (*n* = 9), and rats (*n* = 6). Experimental AC-enriched diets were from as many as 12 sources, i.e., purple/sweet potatoes (*n* = 5), red cabbage (*n* = 1), purple corn (*n* = 1), tart cherries (*n* = 1), blackberries (*n* = 2), blueberries (*n* = 2), bilberries (*n* = 3), strawberries (*n* = 2), black raspberries (*n* = 2), açaí (*n* = 1), grape (*n* = 1), or chokeberries (*n* = 1).

**Table 6A:** Features of included articles reporting role of AC-enriched diet in the CRC prevention in animal models.

Article	Animal model	Subjects n°	Food or extract	Dose of AC	Type of study	Risk reduction
Charepalli et al., 2015	A/J Male mice, AOM-induced CRC	52	Baked purple-fleshed potato (PP) extract	0.14%	Interventional	Reduced incidence of tumors (larger than 2 mm) by 50%
Lippert et al., 2017	Balb/c female mice, AOM/DSS-induced CRC	50	Anthocyanin-rich extract (ARE) obtained from bilberries	Unknown	Interventional	1% ARE-treated mice had 30% tumor number reduction; 10% ARE-treated mice almost no detectable tumors
Lim et al., 2013	CF-1 mice, AOM-induced CRC	30	Anthocyanin-enriched purple-fleshed sweet potato (P40)	0.075%, 0.15%, 0.23%	Interventional	Numbers of large and medium aberrant crypt foci (ACF) were significantly (*p* < 0.01) reduced in mice fed 10%, 20%, or 30% P40. There was no effect of P40 on small ACF formation
Shi et al., 2015	Crj: CD-1 (ICR) male mice, AOM/DSS-induced CRC	50	Lyophilized strawberries	1.5%, 3%, 6%	Interventional	The incidence of tumors decreased by 64%, 75%, and 44%, in groups that have received 2.5%, 5%, and 10% of strawberries
Zhang et al., 2018	C57BL/6J mice, AOM/DSS-induced CRC	30	Anthocyanin extract from black raspberries (BRB)	7 µmol/g/day	Interventional	Mice fed BRB had higher expression of miRNA-24-1-5p
Asadi et al., 2017	C57BL/6J-APC^min/+^ mice	160	Microwave cooked sweet potato (flesh and skin) dietary supplement and AC-enriched extract (ARE) from sweet potato.	0.02%	Interventional	Flesh-, skin-, and ARE-supplemented diets caused significant reduction (*p* < 0.001) in total mean polyp number in small intestine and colon. Supplemented diet did not reduce the number of large polyps (>2 mm) in colon
Kang et al., 2003	Apc^Min^ mice	50	Lyophilized tart cherry	0.08%	Interventional	Mice consuming anthocyanins, cyanidin, or tart cherries had fewer (*P* < 0.05) adenomas in the cecum than mice consuming the control diet or sulindac. The total burden of colonic adenomas was not significant in mice consuming anthocyanins, cyanidin, or tart cherries compared to mice consuming the control diet or sulindac
Bobe et al., 2006	APC ^Min^ mice	141 (74 females e 67 males)	Anthocyanin-rich tart cherry extract (ARE)	0.019%, 0.038%, 0.075%, 0.15%	Intervention	Mouse fed both ARE (at any experimental concentration) and sulindac had 20% less tumor number and 22% lower tumor volume with respect to animals fed sulindac alone in small intestine. There are no data for colon
Cooke et al., 2006	APC^min/+^ mice	148	Mirtoselect containing 40% of anthocyanins from bilberries	0.012%, 0.04%, 0.12% corresponds to 0.36, 1.2, 3,6 mg/mouse	Interventional	Observations in small intestine: dose-dependent decrease in tumor number; at the highest concentration of 0.3% diet supplement, adenoma number decreased by 45% and by 30% for C3G and Mirtoselect, respectively. Few large lesions in colon with nonsignificant reduction after AC treatment
Hagiwara et al., 2001	F344/DuCrj Male rats, DMH/PhIP-induced CRC	84, divided in 6 groups	Purple corn color (PCC)	1.1%	Interventional	Average number of ACF was lower in rats fed PCC (0.8 ± 0.7) with respect to control (2.5 ± 1.8); number of colon adenomas and adenocarcinomas significantly reduced when animals fed PCC.
Harris et al., 2001	Fischer 344, Male rats AOM-induced CRC	216	Lyophilized black raspberries (BRB)	0.05%, 0.1%, 0.2%	Interventional	ACF multiplicity decreased significantly in animals fed BRB. Total tumor multiplicity declined by 42%, 45%, or 71% in groups with diets containing 2.5%, 5%, and 10% BRB. Adenocarcinoma multiplicity reduced by 28%, 35%, and 80% in the 2.5%, 5%, and 10% BRB groups, respectively. Only 10% BRB group was significant (*p* < 0.01); no significant differences in total tumor, adenoma, or adenocarcinoma incidence after BRB consumption
Hagiwara et al., 2002	F344/DuCrj Male rats DMH/PhIP or PhIP-induced CRC	112	Red cabbage color (RCC) Purple sweet potato color (PSPC)	3% 2.5%	Interventional	Significant decrease in ACF number in animals fed RCC but not PSPC. Decrease in adenomas, adenocarcinomas, and average tumor number/rat by AC-rich compounds RCC and PSPC
Lala et al., 2006	Fischer 344 rat males AOM-induced CRC	40	AREs from bilberry (11% ACs); chokeberry (7.7% ACs); grape (14.7% ACs)	26 mg/kg per day	Interventional	Total ACF were reduced (*p* < 0.05) in animals fed AREs. The number of large ACF was also reduced (*p* < 0.05) in bilberry and chokeberry ARE-fed rats. No significant difference (*p* > 0.05) was observed among the small ACF number in animals fed AREs
Fernández et al., 2018	*Rattus norvegicus* F344 male AOM/DSS-induced CRC	30	Dehydrated blackberries and strawberries containing 1.1% (W/W) anthocyanins	0.11% 22 mg/day	Interventional	Reduction in the number of polyps equal to 46.4%; total tumor area not significantly reduced
Fragoso et al., 2018	Wistar rats DMH/TNBS-induced colitis-associated carcinogenesis (CAC)	75	Açaí pulp (AP)	≈2 mg/day	Interventional	Significantly reduced total number of ACF in animals treated with AP; 5% AP treatment caused decreased number of high-grade dysplasia
TABLE 6B |						
Article	Human population/group of people	Subjects n°	Food or extract	Dose of AC	Type of study	Risk reduction
Thomasset et al., 2009	Patients with diagnosed primary adenocarcinoma and others with liver metastases	25 (15 primary + 10 metastases)	Mirtocyan (standardized blueberry extract)	0.5, 1.0, or 2.0 g ACs	Interventional	Tumor tissue proliferation decreased by 7%
Mursu et al., 2008	Finnish men	2,590	Free choice diet	5.9 mg/day	Prospective cohort	Absent
Xu et al., 2016	Cancer patients in Guangzhou, China	1,632 (910 male and 722 female) CRC patients and 1,632 healthy controls	Fruits and vegetables, tea	20.64 mg/day	Retrospective investigative observation study (case–control study)	For fruits and vegetables: borderline OR = 0.80 (95% CI 0.64, 1.00) (*P*_trend_ = 0.08), For tea AC: absent
Nimptsch et al., 2016	Health Professionals Follow-Up Study and Nurses’ Health Study	42,478 male and 76,364 female participants	Free choice diet	15 mg/day	Prospective investigative observational (prospective cohort study)	Absent

Studies were either observational (*n* = 3) or interventional (*n* = 16). The latter were done essentially only in murine species (*n* = 9 in mice; *n* = 6 in rats), whereas only one was in humans. Observational studies recorded AC intake by interviewing subjects. In interventional studies, the orally administered doses of AC ranged from 0.5 to 3 g in humans and 0.03% to 30% dietary supplement in animals. Risk reduction was observed in only one human observational study, though there may be several confounding factors. Intervention studies in animals (*n* = 15) showed, by contrast, effects related to the number of colorectal tumors, their size, total burden, or volume.

### Animals With CRC Induced by Carcinogenic Chemicals

Preclinical *in vivo* experimental data have been obtained from interventions on rodent models of CRC.

Chemically induced carcinogenesis by various combinations of compounds, such as azoxymethane (AOM)/1.2-dimethylhydrazine (DMH), azoxymethane/dextran sodium sulphate (DSS), or 1,2 dimethylhydrazine/2-amino-1-methyl-6-phenylimidazo[4,5-b]pyridine (PhIP), develops mostly in colon and resembles human pathology. Animals usually present histological modification of intestinal tract, like ACF (when used only one carcinogen) or progressive grades of dysplasia and intraepithelial neoplasia (combination of two carcinogens). Disadvantage of these models is a very high exposure to chemicals, which is uncommon for humans. However, they are considered useful tools to test chemopreventive molecules, like AC in colon carcinogenesis (Femia and Caderni, 2008).

### Studies With Mice

Diet with AC-containing purple-fleshed potatoes suppressed the incidence of tumors in the mouse model by elimination of colon cancer stem cells (*in vitro* observation). Furthermore, these animals presented milder signs of gastrointestinal toxicity (e.g., stomach/intestinal ulcers), suggesting that AC treatment may be more effective than the anti-inflammatory drug sulindac (Charepalli et al., 2015).

Anthocyanin-rich extract (ARE) from bilberries, administered as a 10% supplement to regular diet, almost abolished macroscopically visible tumors in AOM/DSS AOM-induced CF-1 mice model. Even when their diet was supplemented with only 1% ARE, tumors were smaller and fewer than that in controls (Lippert et al., 2017).

A supplementary diet with AC-enriched purple-fleshed potato (P40) extract, administered to AOM-induced CF-1 mice model, reduced the total number of large and medium, but not the small, ACF (Lim et al., 2013).

A very detailed study conducted by Shi et al. showed an important contribution of lyophilized strawberries in amelioration of numerous CRC-related parameters. AOM/DSS-induced CD-1 mice developed tumors, whose incidence and multiplicity was decreased significantly when animals were fed strawberries. Besides colorectal tumors, mice experienced an inflammatory response, as shown by increased expression of inflammatory mediators in the colon epithelium. AC-enriched strawberry powder, added to normal diet, interfered and counteracted the pathway of inflammatory mediators. Phosphorylation of pro-inflammatory signaling molecules (PI3K, Akt, ERK1/2, NFκB) was significantly reduced. Consequently, mRNA expression of the inflammatory mediators TNF-α, IL-6, COX-2, and iNOS was down-regulated. As it has been suggested by many studies, end products of arachidonic acid metabolism deriving from the cyclooxygenase pathway (i.e., prostaglandins, PG) can worsen colon cancer prognosis. Indeed, dietary strawberries, (2.5%, 5%, 10% by weight) affected tumor-induced increase of COX-2 and iNOS activity, detected as dose-dependent reduction of PGE2 (30%, 65%, and 70%, respectively), and reduced total nitrate and nitrite production (Shi et al., 2015).

Black raspberry ACs were able to up-regulate miRNA-24-1-5p expression in colon tissue from mice with AOM/DSS-induced CRC. Protective effect of this miRNA was manifested through the inhibition of the cell proliferation-related wnt/β-catenin pathway and consequent attenuation of cancer cell growth, migration, and survival. *In vitro* studies on HCT116 and CaCo-2 cell lines confirmed these observations (Zhang et al., 2018).

### Studies With Rats

The dietary supplement Purple corn color (PCC, containing 21.5% cyanidin 3-glucoside) significantly decreased the number of colonic nodules in F344 rats treated with DMH and PhIP. Hyperplastic and neoplastic lesions were observed also in other organs (jejunum, pancreas, liver, prostate, and seminal vesicle), but their incidences were not affected by PCC administration, showing that the supplement was bioactive only in the colorectal tract. Animal treatment with PhIP alone induced the formation of ACF and their average number was diminished by co-administration of PCC (Hagiwara et al., 2001).

Lyophilized black raspberries (BRB containing 1.7% ACs) caused a limited reduction effect on AOM-induced rat colon carcinogenesis. BRB had the strongest impact on the initial step of carcinogenesis, characterized by ACF formation. The ACF number was significantly reduced at all given BRB concentrations. The total tumor number was also significantly reduced in the BRB-treated animals compared with controls. No significant differences in adenoma incidence or multiplicity were found among groups. The multiplicity of adenocarcinomas was significantly reduced only in the BRB group that received the highest AC supplement, i.e., 10% BRB. Nevertheless, all treatments (2.5%, 5%, and 10% BRB) showed to significantly reduce 8-hydroxy-deoxy guanosine (8-OHdG), a urinary biomarker of oxidative DNA damage, thus showing that BRB could be absorbed and exert wide-ranging antioxidant action *in vivo* (Harris et al., 2001).

Rats treated with AOM to develop CRC lesions were fed with diets enriched in AC from bilberry, chokeberry, or grape. The total number of ACF was significantly reduced with all three AREs. All extracts caused significant reduction of large or medium ACF. However, none of the three extracts had significant effects on small ACF. Rats fed bilberry and grape extracts, but not chokeberry, exhibited lower expression of COX-2 mRNA levels in colonic mucosa. Reduction in urinary 8-OHdG levels was not observed for any treated group, unlike in the above-mentioned case of BRB (Harris et al., 2001). AREs-fed rats had significant increases in fecal moisture content and fecal amount as well as dramatically reduced bile acids (promoters of colon cancer) in fecal extract. Authors stressed that these processes are connected and can contribute to cancer prevention (Lala et al., 2006).

Anthocyanin-rich compounds, such as purple sweet potato color (PSPC) and red cabbage color (RCC), used as 5% supplement in regular diet, ameliorated some of the parameters of DMH- and/or PhIP-induced carcinogenesis in rats. The average number of ACF was significantly decreased in PhIP-treated when they were fed with RCC, but not with PSPC. Rats that were treated by the combination of two carcinogenic agents, such as DMH and PhIP, developed macroscopic colon nodules, mainly distributed in distal colon and rectum. Diets supplemented with PSPC or RCC caused a significant decrease in number of adenomas, adenocarcinomas, and average tumor number per rat (both benign and malignant) (Hagiwara et al., 2002).

The rat model of AOM/DSS-induced carcinogenesis was used to test the effect of AC extracts enriching sausages, so to mimic a diet containing meat. Extracts from strawberries and blackberries ameliorated some histopathological parameters, such as the number of hyperplastic Peyer patches (used as a measure of pro-inflammatory condition). These diets induced the increase of total antioxidant capacity in the blood plasma. The average number of colon polyps was significantly decreased in animals fed AC-rich sausages, suggesting a contribution of AC to protection against CRC. However, no significant reduction of the total tumor area could be observed. Detailed analysis of intestinal microbiota composition (16S ribosomal RNA sequencing) of caecum content revealed the reduced expression of pro-inflammatory *Bilophila wadsworthia* in AC-functional sausage group if compared to control sausage group (Fernández et al., 2018).

The palm tree fruit açaì, growing in South America, has a high content of AC (3.19 mg/g dry weight), with cyanidin 3-glucoside and cyanidin 3-rutinoside as the principal ones (Schauss et al., 2006). Lyophilized açai pulp (AP), as 5% or 7.5% supplement, reduced the total number of ACF in rats with colitis-associated colon carcinogenesis. Treatment with 5% AP decreased tumors with high-grade dysplasia more than tumors with low-grade dysplasia. AP also decreased cell proliferation in tumors (Ki-67 staining) and increased expression of tumor suppressor genes, such as Dlc1, Akt3, and the anti-inflammatory genes Ppara (Fragoso et al., 2018).

### Mice With Hereditary Predilection to CRC

The adenomatous polyposis coli (APC)^min^ mice model, a standard experimental CRC model for preclinical trials, presents a mutation in the murine homolog of the human APC tumor suppressor gene. As a result, mice develop multiple neoplasms predominantly in the small intestine. The total number of adenomatous polyps (particularly those ≤2 mm) in the small intestine significantly decreased when APC^min^ mice were fed with a basic diet added with sweet potato flesh, skin or AC-rich extract from sweet potato. Sweet potato extracts were significantly more efficient in diminishing polyp numbers than the flesh-or skin-supplemented diet. The number of polyps in colon was even increased with skin and ARE-supplemented diets, showing their inability to counteract carcinogenesis (Asadi et al., 2017).

Non-steroidal anti-inflammatory drugs demonstrated to be effective in cancer prevention. Nevertheless, their prolonged use can cause intestinal tract damage. It has been suggested that their combined use with phytochemicals may improve CRC prevention. APC^min^ mice fed with either lyophilized tart cherry ACs (including cyanidin 3-O-sambubioside and other complex glycosides of cyanidin) or pure cyanidin had different responses on tumor frequency and volume, depending on the intestinal tract area. In the caecal part, adenomas were significantly fewer and smaller in animals fed with either extracts or the pure aglycone with respect to mice consuming the control diet or sulindac, a non-steoidal anti-inflammatory drug. The same treatment had no significant effect on colonic tumor volume or number (Kang et al., 2003). A combined diet of AC-rich tart cherry extract with sulindac given to APC^min^ mice resulted in lower tumor number and smaller total tumor area per mouse in small intestine with respect to mice fed sulindac alone. These observations were AC dose-independent. The authors clarified that the equivalent effective dose of AC for humans would be very unusual (more than 1 kg of AC extract/day), so that further tests should determine the lowest effective dose of AC in inhibiting intestinal adenoma development (Bobe et al., 2006).

In the study where APC^min^ mice consumed either Mirtoselect (bilberry extract containing 40% of ACs, such as glucose, galactose, and arabinose conjugates of delphinidin, cyanidin, petunidin, peonidin, and malvidin) or cyanidin 3-glucoside as 0.03%, 0.1%, and 0.3% diet supplements, a dose-dependent reduction of small-size adenoma number was observed. Adenomas were mostly distributed in the medial and distal sections of the small intestine, with very few lesions in colon (Cooke et al., 2006).

### Human Studies

Contrarily to preclinical studies, human studies did not demonstrate encouraging data on the relation between AC-rich diets and prevention of CRC.

In the interventional study performed by Thomasset et al. (2009), a group of 25 CRC patients received an AC-rich extract, mirtocyan (also said Mirtoselect) (0.5–2.0 g/day of AC) for 7 days. The authors had performed preliminary pharmacokinetic studies and identified AC, their methyl and glucuronide metabolites in plasma, colorectal tissue, and urine, but not in the liver. In treated patients, proliferation of cancer tissue was decreased by 7%. In the pharmacodynamic part of the investigation, they found that the concentration of circulating procarcinogenic Insulin-like growth factor 1 (IGF-1) was not significantly reduced. Mirtocyan did not affect the levels of oxidative DNA damage biomarkers.

A prospective cohort study on middle-aged Finnish men, with mean flavonoid intake of 131.0 ± 214 mg/day, demonstrated no correlation between flavonoid consumption and CRC risk reduction. Anthocyanidins represented 5% (5.9 mg/day) of all flavonoids in food (Mursu et al., 2008).

A retrospective case-control study was performed by including both CRC patients and healthy controls. Eating habits were recorded through. The authors of this study found only borderline significant inverse association between flavonoids/AC (from fruits and vegetables) intake and colon cancer risk increase (Xu et al., 2016).

A huge prospective cohort study with 2519 registered CRC cases found no correlation between high daily flavonoid (4% AC) intake and risk of CRC. Dietary intake data were collected every 4 years through food-frequency questionnaire (Nimptsch et al., 2016).

### AC-Related Anti-Cancer Molecular Mechanisms

We have retrieved 10 articles reporting molecular mechanisms of AC-related anticancer activity. **Table 7** summarizes features that give information related to this topic. Most of the studies (*n* = 8) were performed *in vitro* on cell models; only one was performed *in vivo*. Early-stage colon cancer cells HCT-116 were used in five studies, whereas four studies used cells associated with advanced stage colon cancer, such as HT-29. Two studies reported data obtained from Caco-2 cells, regarded as a model of differentiated intestinal epithelium; one study used non-cancer colon fibroblast cells, i.e., CCD-18Co. The pure AC molecules used to test cancer-preventive molecular mechanisms were cyanidin (*n* = 1), cyanidin-3-glucoside (*n* = 3), cyanidin chloride (*n* = 1), delphinidin (*n* = 2), delphinidin-3-O-glucoside (*n* = 1), or AC-rich extracts from grapes (*n* = 2), cocoplum fruit (*n* = 1), and bilberries/blackcurrant (*n* = 1).

**Table 7:** Features of included articles reporting AC-related anticancer molecular mechanism.

Experimental model								
Article	Animals (*n* of subjects)	Cells	Single AC molecules or pure mixtures thereof	[AC]	IC_50_	Reaction, pathway or function modified by AC	Molecular target	Molecular marker
Cooke et al., 2006	APC^min/+^ mice (148 animals)		Cyanidin-3-glucoside purified from blackberries ∼0.9, 3 or 9 mg per mouse per day	0.3 mg/ml, 1 mg/ml, or 3 mg/ml in diet		45% reduction of adenoma numbers		
Mazewski et al., 2018		HT-29 and HCT 116	Cyanidin-3-O-glucoside (C3G) Delphinidin-3-O-glucoside (D3G)	0.01, 0.1, 1, e 10 µM	0.10 µM 2.37 µM	***EGFR kinase inhibition	EGFR	
Briviba et al., 2001		HT-29	Cyanidin	10 µM and 100 µM		Neurotensin and EGF-induced intracellular Ca^2+^ concentration and cell proliferation		
Jang et al., 2008		HT-29 e HCT-116	Delphinidin	25, 50, 100 µM		Oxidative stress		Phosfoglycerate kinase—PGK1 expression attenuated by delphinidin
Renis et al., 2008		Caco-2	Cyanidin chloride (CY) and Cyanidin-3-O-β glucopyranoside (CY3G)	25, 50, 100, 200 µM		DNA fragmentation; cell cycle: ****ATM, p53 e p21, topoisomerase IIβ; DNA damage: HSP70 e OGG1, p21 e p53	DNA	
Shin et al., 2009		HCT-116	*AIMs	5, 15, 30, 45, 60 µg/ml;	<60 µg/ml	Apoptosis: caspase-3, caspase-8, caspase-9; XIAP, cIAP-1, cIAP-2, Bcl-2, Bax, Bcl-XL, Bid, of poly (ADP-ribose) polymerase (PARP), p38-MAPK-JNK-ERK (pathway); PI3k/Akt pathway; XIAP		
Yun et al., 2009		HCT-116	Delphinidin	30, 60, 120, 180, 240 µM	110 µM	Apoptosis: PARP (is cleaved), Caspase-3, caspase-8, caspase 9, Bax (increase), Bcl-2 (decrease), IKKα (inhibition) IκBα, Cyclin B, cdc2, p21^WAF1/Cip1^ and p53, NF-κB signaling		
Shin et al., 2011		HCT-116	*AIMs	5, 15, 30, 45 µg/ml;		Tight junctions modification: Claudine (1-3-4-5); Matrix metalloproteinase: MMP-2 and -9. p38-MAPK and PI3K/Akt signaling pathway		
Venancio et al., 2017		HT-29 CCD-18Co	**CP anthocyanins	5, 10, 20 µM		Inflammation: NF-κB1, TNF-α, IL-1β e IL-6		
Anwar et al., 2016		Caco-2 NIH/3T3	AC (96%) extract from bilberries and blackcurrant	62.5250 µg/ml		Apoptosis: caspase -3 Cell cycle: p21^Waf1/Cip1^ Oxidative status: ROS		

*AIMs from Vitis coignetiae Pulliat (Crimson glory vine). Composition: delphinidin-3,5- diglucoside; cyanidin-3,5-diglucoside; petunidin-3,5- diglucoside; delphinidin-3-glucoside; malvdin-3,5- diglucoside; peonidin-3,5-diglucoside; cyanidin-3-glucoside; petunidin-3-glucoside; peonidin-3-glucoside; malvidin-3-glucoside.

**CP anthocyanins from Crysobalanus icaco L. Delphinidin-3-glucoside (1,162 µg ml^−1^), Cyanidin 3-glucoside (382 µg ml^−1^), Petunidin 3-glucoside that coeluted with either delphinidin 3-(6″-acetoyl) galactoside or delphinidin 3-(6″- oxaloyl) arabinoside (1,396 µg ml^−1^), Peonidin 3-glucoside (345 µg ml^−1^), Petunidin 3-(6″-acetoyl) galactoside or Petunidin 3-(6″-oxaloyl) Arabinoside (611 µg ml^−1^) and Peonidin 3-(6″- acetoyl) glucoside or Peonidin 3-(6″-oxaloyl) arabinoside (689 µg ml^−1^).

***Epidermal growth factor receptor.

****Ataxia-Telangiesctasia mutated gene.

Concentrations of AC ranged from 0.01 µM to 240 µM or 5 to 250 µg/ml. Studies showed mostly positive actions of AC on processes involved in carcinogenesis, like apoptosis, inflammation, proliferation, tight junction modulation, oxidative stress, and tumor number.

Phenolic-rich extracts, rather than AC alone, were able to inhibit proliferation of human colon cancer cell lines, such as HT-29 and HCT-116, pointing to the existence of potential synergic actions of different polyphenols (Mazewski et al., 2018). On the other hand, the same authors concluded that two pure AC molecules, i.e., cyanidin 3-O-glucoside and delphinidin 3-O-glucoside, were more efficient than phenolic-rich extracts as inhibitors of EGFR tyrosine kinase. By targeting this receptor, both molecules showed to interfere with the delicate balance of two opposed processes, like cancer cell survival and programmed cell death. Cyanidin 3-glucoside was also more efficient in reducing adenoma numbers in small intestine than the AC-rich bilberry extract (Cooke et al., 2006).

Cancer cells have both a huge proliferative potential and impaired mechanisms of apoptosis. Some authors (Yun et al., 2009) have suggested that targeting these pathways can be promising for the development of new anticancer drugs. They examined the mechanism(s) by which the delphinidin aglycone is able to induce apoptosis in HCT-116 cell line. Delphinidin treatment induced activation of caspases and PARP cleavage, two authentic mechanisms of apoptotic response. Delphinidin treatment resulted in a significant increase in proapoptotic protein Bax and decrease in antiapoptotic protein Bcl-2, leading to cell cycle arrest. Delphinidin also upregulated the expression of both the tumor suppressor protein p53 and its downstream target p21. It caused downregulation of both cyclin B1 and cdc2, again leading to cell cycle arrest. They found that delphinidin treatment of HCT-116 caused downregulation of the NF-κB pathway, as shown by decreased expression of the p65 subunits of NF-κB, reduced IκB phosphorylation, and inhibited nuclear translocation of NF-κB.

In studies on the human colon cancer HT-29 cell line, some authors (Jang et al., 2008) suggested PGK1 as a potential biomarker of intracellular oxidative damage. Cells exposed to 50 µM H_2_O_2_ for 24 h showed significant expression of PGK1. Interestingly, cells co-treated with delphinidin had attenuated expression of this protein. High levels of PGK1 are associated with tumor survival and angiogenesis. These authors suggested that the antioxidant potential of delphinidin could contribute to anti-cancer strategy.

The apoptotic signaling pathway was also explored in HCT-116 cells (Shin et al., 2009). An AC mix, isolated from grape (AIMs), was used in relatively low concentrations (5–60 µg/ml), easily reachable *in vivo*. The authors observed decreased expression of pro-caspase-3, -8, and -9, activation of caspase-3 and PARP cleavage. AIMs reduced the expressions of anti-apoptotic proteins (XIAP, cIAP-1, and cIAP-2), so promoting cell death. They suggested that suppression of XIAP was mediated by suppression of the PI3K/Akt pathway. Other apoptotic pathways were influenced by AIMs through increased p38-MAPK phosphorylation and attenuated JNK phosphorylation.

Another AC mixture derived from bilberries and blackcurrant exhibited anti-proliferative effects in Caco-2 cells, by increasing both pro-apoptotic caspase-3 and the cell cycle-related protein p21^Waf/Cip1^ (Anwar et al., 2016). Furthermore, this AC extract caused accumulation of ROS in Caco-2 cells but not in normal fibroblast cells (NIH/3T3), thus demonstrating potentially selective cytotoxicity toward cancer cells.

Some authors suggested that the chemical structure of AC could strongly influence their biological function (Renis et al., 2008). They used human colon carcinoma CaCo-2 cells and two ACs, i.e., cyanidin 3-O-glucoside (CY3G) and its aglycon cyanidin chloride (CY), to examine if the substitution pattern of the β-ring could influence AC-mediated cell growth signaling. Reactive oxygen species (ROS) were found to be elevated in almost all cancers. Cancer cells balance ROS levels with anti-oxidative enzymes levels in favor of their own proliferation and survival. CY (25 µmol/L) was more successful in decreasing ROS level than CY3G. The typical Comet assay showed that DNA fragmentation occurred only at moderate (25–100 µmol/L) and high concentrations of CY3G (200 µmol/L). Possible intercalation of CY3G and CY molecules to DNA was explored in the atypical Comet assay performed on naked DNA; this time, dose-dependent fragmentation was observed at low levels of CY3G (25 or 50 µmol/L). In the absence of DNA repair mechanisms, CY-caused damage was even more dramatic, whereas CY3G caused damage only at high concentration (200 µmol/L). CY3G was more efficient than CY in counteracting the H_2_O_2_-induced DNA damage. Specific mutagenic lesions occurring after exposure to ROS were removed by 8-oxoguanine DNA glycosylase (OGG1) (a DNA repair enzyme). Expression of OGG1 was decreased after cell treatment with 25 µmol/L CY and increased at 200 µmol/L. On the contrary, CY3G treatment-induced OGG1 expression at the low concentration of 25 µmol/L. Heat shock proteins (HSP70) are highly expressed in CaCo-2 cells and their expression was even more increased when the cells were treated with CY3G or CY. The same pattern of increased expression of cell cycle-related proteins, such as Ataxia telangiectasia mutated gene (ATM), p53, and topoisomerase IIβ, was obtained by both CY3G and CY. The authors have proposed an explanation, i.e., that the increase in ATM triggers expression of mutated form of p53, not capable to act as a transcriptional factor for p21.

Metabolic alterations in cancer cells can lead to high metabolic activity and increased proton production. Briviba et al. (2001) demonstrated that growth factors, neurotensin and EGF could increase proton extrusion, measured as extracellular acidification, in the human cancer cell line HT-29. Proton extrusion is proposed to be involved in cell growth (Di Sario et al., 1999). This process was inhibited by cyanidin (10 µM), but by none of its glycosides, i.e., cyanin (i.e., cyanidin 3,5-O-diglucoside) and idaein (i.e., cyanidin 3-galactoside). Neurotensin-induced [Ca^2+^]i was significantly lower in cells pre-incubated with 10 µM cyanidin. Cyanidin did not change extracellular-signal-regulated kinase (ERK) phosphorylation. The suggested role of cyanidin is the modulation of intracellular signaling between receptors and [Ca^2+^]i, consequently affecting cellular metabolism and growth. Glucosidase, released by intestinal bacteria, could hydrolyze ACs to aglycones, able to inhibit [Ca^2+^]i and cellular metabolism induced by neurotensin and EGF.

Cocoplum anthocyanins (CP) have been demonstrated to exercise anti-inflammatory activity in both human cancer cells HT-29 and inflamed benign cells (Venancio et al., 2017). Inflammation is closely related with carcinogenesis. Persistent inflammation favors growth and inhibition of apoptosis in transformed cells. CCD-18Co colon fibroblasts were selected for their low levels of ROS. Thus, experimental induction of inflammation could be monitored by measurable intracellular increases of ROS. TNF-α-induced inflammation (measured as ROS production) in these non-malignant colon fibroblasts was significantly decreased with CP extract. After this treatment, NF-κB1 mRNA expression was decreased with CP extract, along with protein expression of TNF-α, IL1β, and IL-6. The authors also reported decreased mRNA expression of inflammatory mediators (NF-κB1, TNF-α, IL1β, and IL-6) in HT-29 colon cancer cells after CP treatment, whereas protein expression decrease was observed only for TNF-α.

In the studies conducted in HCT-116 (Shin et al., 2011), AC isolated mix (AIM) from the Asiatic grape variety *V. coignetiae* Pulliat (45 µg/ml) diminished the expression of the tight junction (TJ) proteins known as claudins, as well as several matrix metalloproteinases. Preservation of TJ integrity and inhibition of proteolytic digestion of the extra cellular matrix is important to prevent tumor cell mobility and invasiveness. In functional assays, anthocyanins AC also exerted potential anticancer activity. The AC mix increased TJs tightening, measured as transepithelial electrical resistance. The same AIM concentration was successful in diminishing the HCT-116 cells capability to penetrate ECM. They proposed that AIM-induced down regulation of MMP-2 and -9, as well as of claudin-3, can be partially attributed to the p38-MAPK activation and the suppression of the PI3K/Akt pathway.

## Discussion

**Figure 2:**
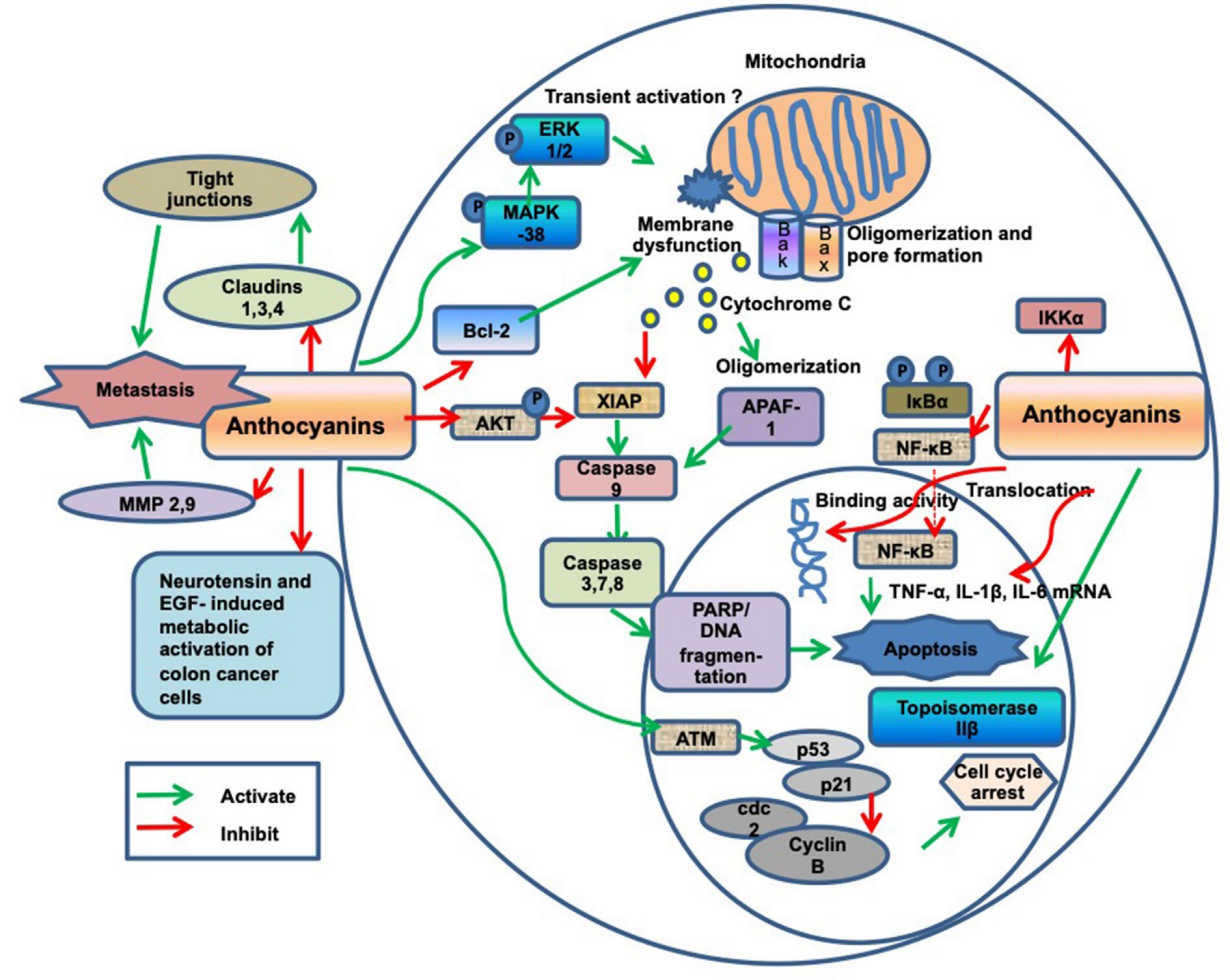
Diagram of anthocyanin (AC)-related anti-cancer molecular mechanisms.

Concerning the general question whether AC prevent or not CRC *in vivo*, the detailed analysis of the literature enabled to provide some answer to the initially defined questions, as follows:

1. *Are there any studies performed on human groups or populations?* We were able to identify four studies involving human subjects: one interventional study (Thomasset et al., 2009), one retrospective cohort study (Xu et al., 2016), and two prospective cohort studies (Mursu et al., 2008; Nimptsch et al., 2016). A synopsis of these studies is presented in **Table 8**.
  The reported effects of AC were, however, controversial. In fact, the single interventional study showed borderline data, showing that AC administration mildly affected tumor cell proliferation (7% decrease) only at the lowest dose of mirtocyan. No dose dependence could be found, since, two higher doses gave no significant decrease. Other carcinogenesis-related markers, such as serum IGF-I levels, oxidative DNA damage or apoptotic index, were not affected. The authors recognized that they tried elevated doses of AC, 0.5 to 2.0 g/day, corresponding to unusually high servings, ranging 350 to 1,500 g fresh bilberries. In three cohort studies, diets included several flavonoid classes, with an estimated consumption of AC of no more than 6 to 20 mg/day. While one of these studies (Xu et al., 2016) found that CRC prevention correlated with the intake of flavonoids (including AC) in fruits and vegetables, no specific AC-related effect was reported. Two prospective cohort studies found no AC effect. Taken together, these studies showed weak or no correlation between AC intake and CRC risk decrease.
2. *Are there any studies performed on animals? What animal models have been used?* As many as 15 intervention studies tested the role of AC in CRC prevention in different murine strains, i.e., four rat strains (F344/DuCrj, Fischer 344, Sprague–Dawley, *Rattus norvegicus* F344) and four mouse strains (Apc^Min^, A/J, Balb/c, Crj: CD-1). Animals were fed with standard rodent food in combination with supplements enriched with AC extracts or fruits. The AC supplementation ranged from µg to g. In some studies, though, the doses of AC consumed by animals could not be calculated. A synopsis of these studies is in **Table 9**.
  With the exception of the mouse Apc^Min^ model (a knockout for the human homologue of the tumor suppressor APC gene), all murine strains were investigated after chemical induction of CRC lesions of different tumor stages. In seven studies, AC treatments were more efficient on ACF rather than on developed cancers. In the genetic model Apc^Min^, adenomas/adenocarcinomas were mostly developed in the small intestine than in the colon, where they were very rare, making this model not perfectly representing CRC in humans. Here, four studies showed that the preventive effect of AC was predominantly on adenomas rather than on adenocarcinomas, suggesting that AC may be most effective at early tumor stages. These animal models have both advantages for short-term pre-clinical screening goals and disadvantages related to differences in lifespan, the diet and environmental exposure of human and murine species. Thus, experimental design in murine models of induced CRC and interpretation of results must take these differences in due consideration (Femia and Caderni, 2008).
  Concerning the molecular mechanisms whereby AC prevent CRC *in vivo*, the analysis of the included articles enabled to give answers to our research interests, as follows:
3. *Are there any studies performed in cellular models?* Only nine articles complying with the inclusion criteria reported *in vitro* studies on the molecular mechanisms by which AC inhibit CRC progression, as outlined in **Table 10**.
  Numbers of articles were excluded (*n* = 57), because using heterogeneous polyphenolic extracts, instead of pure molecules. All studies were conducted on human colon cancer cells, such as HCT-116 (*n* = 4), HT-29 (*n* = 3), and Caco-2 (*n* = 2). HCT-116 cells, as a model of early stage colon cancer, were used for studies of proliferation, apoptosis and tight junction modifications. HT-29 cells, as a model of colorectal adenocarcinoma, were used in studies of metabolic activation, inflammatory processes, and intracellular oxidative status. Caco-2 cells, colorectal adenocarcinoma cells, were used in studies of cell cycle/stress-related protein expression, DNA damage, proliferation and intracellular ROS level. Only three studies have used normal benign cells, like the human colon fibroblast lines CCD-33Co, CCD-18Co, and the mouse fibroblasts NIH/3T3. These cells were used to test AC cytotoxicity and inhibition of basal cellular ROS production.
4. *What molecules have been used to test anti-cancer effects?* As shown in **Table 10**, six pure molecules were tested, i.e., cyanidin, cyanidin 3-glucoside and cyanidin 3,5-diglucoside, cyanidin 3-galactoside, cyanidin chloride, and delphinidin. Furthermore, four AC mixtures with purity >90% were used.
5. *Which biological processes have been investigated?* AC were shown to alter several fundamental processes that play a key role in cell cycle control and carcinogenesis, as listed in **Table 11** and illustrated in **Figure 2**.
6. *Have specific molecular targets or markers been identified?* Only two studies assessed the direct interaction of AC molecules with molecular targets, such as EGFR (Mazewski et al., 2018) and DNA (Renis et al., 2008), as shown in **Table 7**. By contrast, all the other studies focused on downstream effects, mostly assessed as changes in the levels of signaling components regarded as markers of AC-dependent effects. By this approach, it is however impossible to infer the primary cause of molecular marker changes, because of cross talk involving several signaling pathways. Only one marker was described as being in straight correlation with delphinidin treatment of cancer cells, i.e., phosphoglycerate kinase PGK1 (Jang et al., 2008).

**Table 8:** Human populations or groups surveyed for assessing the relationship linking AC intake to CRC.

Population	Study type	Diet	AC daily dose	Association between anthocyanin intake and CRC risk	Reference
Patients with diagnosed adenocarcinoma and others with liver metastases	Interventional		0.5, 1.0, or 2.0 g	Partial (small effect on cancer cell proliferation) Tumor tissue proliferation decreased by 7%	(Thomasset et al., 2009)
Finnish men cohort	Observational	Scandinavian	5.9 mg	No	(Mursu et al., 2008)
Patients with histologically confirmed colorectal cancer in Guangzhou, China	Observational	Asian	20.64 mg	Partial (related to fruit and vegetable but not tea flavonoids) For fruits and vegetables: borderline OR = 0.80 (95% CI 0.64–1.00) (*P*_trend_ = 0.08), For tea AC: absent	(Xu et al., 2016)
Paramedics (dentists, ophthalmologists, osteopaths, podiatrists, pharmacists, veterinarians) and infertile women	Observational	North American	15 mg	No	(Nimptsch et al., 2016)

**Table 9:** List of animal models used in intervention studies for assessing the relationship linking AC intake to CRC.

Species	Diet	Supplement	AC dose	Effect	References
A/J Male mice, AOM-induced CRC	AIN-93G diet	Baked purple-fleshed potato (PP) extract	0.14%	Reduced incidence of tumors (larger than 2 mm) by 50%	Charepalli et al., 2015
Balb/c female mice, AOM/DSS-induced CRC	Standard rodent diet	Anthocyanin-rich extract (ARE) obtained from bilberries	Unknown	1% ARE-treated mice had 30% tumor number reduction; in 10% ARE-treated mice almost no detectable tumors	Lippert et al., 2017
CF-1 mice, AOM-induced CRC	AIN-93M basal diet	Anthocyanin-enriched purple-fleshed sweet potato (P40)	0.075%, 0.15%, 0.23%	Number of large and medium aberrant crypt foci (ACF), was significantly (*p* < 0.01) reduced in mice fed 10%, 20% or 30% P40. There was no effect of P40 on small ACF formation	Lim et al., 2013
Crj: CD-1 (ICR) male mice, AOM/DSS-induced CRC	AIN-76A diet	Lyophilized strawberries	1.5%, 3%, 6%	The incidence of tumors decreased by 64%, 75%, and 44%, in groups that have received 2.5%, 5%, and 10% of strawberries	Shi et al., 2015
C57BL/6J mice, AOM/DSS-induced CRC		Anthocyanin extract from black raspberries (BRB)	7 µmol/g/day	Mice fed BRB had higher expression of miRNA-24-1-5p in colon tissue	Zhang et al., 2018
C57BL/6J-APC^min/+^ mice	AIN-76 diet	Microwave cooked sweet potato (flesh and skin) dietary supplement and AC-enriched extract (ARE) from sweet potato.	0.02%	Flesh-, skin-, and ARE-supplemented diets caused significant reduction (*p* < 0.001) in total mean polyp number in small intestine and colon. Supplemented diet did not reduce the number of large polyps (>2 mm) in colon	Asadi et al., 2017
Apc^Min^ mice	Modified AIN 93G diet	Tart cherry lyophilized anthocyanins	0.08%	Mice consuming anthocyanins, cyanidin, or tart cherries had fewer (*P* < 0.05) adenomas in the caecum than mice consuming the control diet or sulindac. The total burden of colonic adenomas was not significant in mice consuming anthocyanins, cyanidin, or tart cherries compared to mice consuming the control diet or sulindac	Kang et al., 2003
APC ^Min^ mice	AIN-93G standard diet	Anthocyanin-rich tart cherry extract (ARE)	0.019%, 0.038%, 0.075%, 0.15%	Mouse fed both ARE (at any experimental concentration) and sulindac had 20% less tumor number and 22% lower tumor volume with respect to animals fed sulindac alone in small intestine. There are no data for colon	Bobe et al., 2006
APC^min/+^ mice	AIN 93G standard rodent diet	Mirtoselect containing 40% of anthocyanins, or cyanidin-3-glucoside (C3G) purified from blackberries	0.012%, 0.04%, 0.12% corresponds to 0.36, 1.2, 3.6 mg/mouse	Observations in small intestine: dose-dependent decrease in tumor number; at the highest concentration of 0.3% diet supplement, adenoma number decreased by 45% and by 30% for C3G and Mirtoselect, respectively. Few large lesions in colon with nonsignificant reduction after AC treatment	Cooke et al., 2006
F344/DuCrj Male rats, DMH/PhIP-induced CRC	Commercial Labo MR rodent diet	Purple corn color (PCC)	1.1%	Average number of ACF was lower in rats fed PCC (0.8 ± 0.7) with respect to control (2.5 ± 1.8); number of colon adenomas and adenocarcinomas significantly reduced when animals fed PCC.	Hagiwara et al., 2001
Fischer 344, Male rats AOM-induced CRC	AIN-76A purified rodent diet	Lyophilized black raspberries (BRB)	0.05%, 0.1%, 0.2%	ACF multiplicity decreased significantly in animals fed BRB. Total tumor multiplicity declined by 42%, 45%, or 71% in groups with diets containing 2.5%, 5%, and 10% BRB. Adenocarcinoma multiplicity reduced by 28%, 35%, and 80% in the 2.5%, 5%, 10% BRB groups, respectively. Only 10% BRB group was significant (*p* < 0.01); no significant differences in total tumor, adenoma, or adenocarcinoma incidence after BRB consumption	Harris et al., 2001
F344/DuCrj Male rats DMH/PhIP or PhIP-induced CRC	Commercial Labo MR rodent diet	Red cabbage color (RCC) Purple sweet potato color (PSPC)	3% 2.5%	Significant decrease in ACF number in animals fed RCC but not PSPC. Decrease in adenomas, adenocarcinomas, and average tumor number/rat by AC-rich compounds RCC and PSPC	Hagiwara et al., 2002
Fischer 344 rat males AOM-induced CRC	AIN-93 rodent diet	AREs from bilberry (11% AC); chokeberry (7.7% ACs); grape (14.7% AC)	26 mg/kg per day	Total ACF were reduced (*p* < 0.05) in animals fed AREs. The number of large ACF was also reduced (*p* < 0.05) in bilberry and chokeberry ARE-fed rats. No significant difference (*p* > 0.05) was observed among the small ACF number in animals fed AREs	Lala et al., 2006
*Rattus norvegicus* F344 male AOM/DSS-induced CRC	Protein rodent maintenance diet	Dehydrated blackberries and strawberries containing 1.1% AC	0.11% 22 mg/day	Reduction in the number of polyps equal to 46.4%; total tumor area not significantly reduced	Fernández et al., 2018
Wistar rats DMH/TNBS-induced colitis-associated carcinogenesis	Cereal-based commercial rodent diet	Açaí pulp	≈2 mg/day	Significantly reduced total number of ACF in animals treated with AP; 5% AP treatment caused decreased number of high-grade dysplasia	Fragoso et al., 2018

**Table 10:** List of experimental models used to identify the molecular mechanisms of CRC prevention by AC (not diets or not supplements).

Experimental models	Control cells	Anthocyanins	References
HCT-116	CCD-33Co (colon fibroblast)	Cyanidin-3-O-glucoside Delphinidin AIMs from grape AIMs from grape	Mazewski et al., 2018 Yun et al., 2009 Shin et al., 2009 Shin et al., 2011
HT-29	CCD-18Co (colon fibroblast)	Cyanidin-3-O-glucoside Delphinidin Cyanidin Cyanidin-3,5-di-O-glucoside Cyanidin 3-galactoside Cocoplum anthocyanins	Mazewski et al., 2018 Jang et al., 2008 Briviba et al., 2001 Briviba et al., 2001 Briviba et al., 2001 Venancio et al., 2017
Caco-2	NIH/3T3 (mouse fibroblasts)	Cyanidin-3-O-β glucopyranoside Cyanidin chloride AC mix derived bilberries and blackcurrant	Renis et al., 2008 Renis et al., 2008 Anwar et al., 2016

**Table 11:** List of biological processes (reactions, pathways, or functions) modified by AC.

Biological process	Measurement of effect	Compound	Reference
Oxidative stress	Suppression of **PGK1** (oxidative stress marker) expression	Delphinidin	Jang et al., 2008
	Decrease in **ROS** level	Cyanidin-3-O-glucoside	Renis et al., 2008
	Decrease in oxidative **DNA** damage (Comet assay); Increase in **HSP70**; Dose dependent increase/decrease in **OGG1**	Cyanidin-3-O-glucoside and cyanidin chloride	Renis et al., 2008
	Increase in **ROS**, induction of ROS accumulation	AC mix derived bilberries and blackcurrant	Anwar et al., 2016
Cell cycle-related protein modification	Increase in **ATM, p53** (inactive form) and **topoisomerase IIβ**	Cyanidin-3-O-glucoside and cyanidin chloride	Renis et al., 2008
	Decrease in **cyclin B1** and **cdc2**; Increase in **p53** and **p21**; Suppression of **NF-κB** pathway; inhibition of **IKKα** activation, phosphorylation and degradation of **IκBα**, **p-** **NF-κB/p65**	Delphinidin	Yun et al., 2009
	Increase in **p21** **^Waf/Cip1^**	AC mix derived bilberries and blackcurrant	Anwar et al., 2016
Cancer cell metabolism	Inhibition of neurotensin- and EGF-induced increased rate of extracellular acidification (cellular metabolism) by decreasing **[Ca** **^2+^** **]i**	Cyanidin	Briviba et al., 2001
Inflammation	Decreased mRNA expression of **TNF-α, IL-1β, IL-6, NF-κB1;** Decreased protein expression of **TNF-α, IL-1β, IL-6**	Cocoplum fruit AC extract	Venancio et al., 2017
Cancer cell proliferation	Inhibition of EGF-induced **cell growth**	Cyanidin	Briviba et al., 2001
	Inhibition of **NF-κB DNA-binding activity**	Delphinidin	Yun et al., 2009
ECM modification/angiogenesis and metastasis	Decreased expression of **claudins 1, 3, 4**; **MMP-2 and -9**; increase in transepithelial electrical resistance (tight junction modification)	AIMs from *Vitis coignetiae* Pulliat	Shin et al., 2011
	Inhibition of **EGFR tyrosine kinase**	Cyanidin-3-O-glucoside Delphinidin-3-O-glucoside	Mazewski et al., 2018
Cancer cell apoptosis	Decreased expression of **pro-caspase-3, -8, and -9**; Decreased expression of **Akt, XIAP, cIAP-1, cIAP-2;** Activation of p38-MAPK	AIMs from *Vitis coignetiae* Pulliat	Shin et al., 2009
	Activation of caspases; **PARP** cleavage; increase in **Bax** expression; decrease in **Bcl-2** expression	Delphinidin	Yun et al., 2009
	Increase in pro-apoptotic **caspase-3** level	AC mix derived bilberries and blackcurrant	Anwar et al., 2016
Tumor number	Decrease in adenoma number	Cyanidin-3-O-glucoside	Cooke et al., 2006

Key words are in bold.

## Conclusions

With this systematic review we aimed at bringing to light the actual state of the art in the research exploring AC-specific CRC prevention and the underlying oncotargets. AC have shown to protect against early-stage cancer lesions in experimental animals, but not in humans. Several studies in CRC cell models have shown AC to affect cellular processes related to cell cycle and transformation. However, these effects cannot yet be ascribed to AC-specific molecular interactions with CRC oncotargets, but rather to complex effects resulting in preventing intestinal cells from entering in the cell fate of neoplastic transformation.

We believe that this overview may serve the scientific community to properly select the experimental models and conditions to further clarify the mode of action of AC and their degradation products in modulating the carcinogenetic process in CRC.

## Data Availability Statement

Publicly available datasets were analyzed in this study. This data can be found here: https://www.ncbi.nlm.nih.gov/pubmed/?term=Colorectal+cancer+AND+Anthocyanins.

## Author Contributions

SP designed the method, created and managed the database of the PubMed search output. NM carried out all phases of the systematic review. Both NM and FT independently screened and assessed the included articles. NM and SP drafted the article.

## Funding

This work is an activity of the standard project Agrotur II (code 1473843258), funded by Interreg Italy-Slovenia 2014-2020 (European Regional Development Fund and national funds). The activity goal is to improve knowledge on the health effects of grape antioxidants.

## Conflict of Interest Statement

The authors declare that the research was conducted in the absence of any commercial or financial relationships that could be construed as a potential conflict of interest.
