# Supplementary material for: Anthocyanins in Colorectal Cancer Prevention. A Systematic Review of the Literature in Search of Molecular Oncotargets
Source: Front Pharmacol. 2019 Jun 21;10:675. doi: 10.3389/fphar.2019.00675 (PMC6597886; doi:10.3389/fphar.2019.00675)
Supplement: Supplementary file 1 [file DataSheet_1.pdf]

SUPPLEMENTARY MATERIAL

Table 1S | List of articles excluded after pre-screening with specific motivations.

N°	DOCUMENTS	MOTIVATION
1)	De Sousa Moraes LF, Sun X, Peluzio M do CG, Zhu M-J. Anthocyanins/anthocyanidins and colorectal cancer: What is behind the scenes? Crit Rev Food Sci Nutr. England; 2017;1–13.	Review
2)	Pan P, Lam V, Salzman N, Huang Y-W, Yu J, Zhang J, et al. Black Raspberries and Their Anthocyanin and Fiber Fractions Alter the Composition and Diversity of Gut Microbiota in F-344 Rats. Nutr Cancer. United States; 2017;69:943–51.	Indirectly linked to CRC
3)	Yang N, Sampathkumar K, Loo SCJ. Recent advances in complementary and replacement therapy with nutraceuticals in combating gastrointestinal illnesses. Clin Nutr. England; 2017;36:968–79.	Review
4)	Bishayee A, Haskell Y, Do C, Siveen KS, Mohandas N, Sethi G, et al. Potential Benefits of Edible Berries in the Management of Aerodigestive and Gastrointestinal Tract Cancers: Preclinical and Clinical Evidence. Crit Rev Food Sci Nutr. England; 2016;56:1753–75.	Review
5)	Nunez-Sanchez MA, Gonzalez-Sarrias A, Romo-Vaquero M, Garcia-Villalba R, Selma M V, Tomas-Barberan FA, et al. Dietary phenolics against colorectal cancer--From promising preclinical results to poor translation into clinical trials: Pitfalls and future needs. Mol Nutr Food Res. Germany; 2015;59:1274–91.	Review
6)	Sehitoglu MH, Farooqi AA, Qureshi MZ, Butt G, Aras A. Anthocyanins: targeting of signaling networks in cancer cells. Asian Pac J Cancer Prev. Thailand; 2014;15:2379–81.	Review
7)	Alvarez-Suarez JM, Dekanski D, Ristic S, Radonjic N V, Petronijevic ND, Giampieri F, et al. Strawberry polyphenols attenuate ethanol-induced gastric lesions in rats by activation of antioxidant enzymes and attenuation of MDA increase. PLoS One. United States; 2011;6:e25878.	Not linked to CRC
8)	Russell W, Duthie G. Plant secondary metabolites and gut health: the case for phenolic acids. Proc Nutr Soc. England; 2011;70:389–96.	Gut health
9)	Brown EM, Gill CIR, McDougall GJ, Stewart D. Mechanisms underlying the anti-proliferative effects of berry components in in vitro models of colon cancer. Curr Pharm Biotechnol. Netherlands; 2012;13:200–9.	Review
10)	Olejnik A, Tomczyk J, Kowalska K, Grajek W. The role of natural dietary compounds in colorectal cancer chemoprevention. Postepy Hig Med Dosw (Online). Poland; 2010;64:175–87.	Review<br><br>Not in English

11)	Forester SC, Waterhouse AL. Metabolites are key to understanding health effects of wine polyphenolics. J Nutr. United States; 2009;139:1824S–31S.	Review
12)	Galvano F, Salamone F, Nicolosi A, Vitaglione P. Anthocyanins-based drugs for colon cancer treatment: the nutritionist's point of view. Cancer Chemother. Pharmacol. Germany; 2009. p. 431–2.	Letter to the Editor
13)	Thomasset S, Teller N, Cai H, Marko D, Berry DP, Steward WP, et al. Do anthocyanins and anthocyanidins, cancer chemopreventive pigments in the diet, merit development as potential drugs? Cancer Chemother Pharmacol. Germany; 2009;64:201–11.	Commentary
14)	Farzaei MH, El-Senduny FF, Momtaz S, Parvizi F, Iranpanah A, Tewari D, Naseri R, Abdolghaffari AH, Rezaei N. An update on dietary consideration in inflammatory bowel disease: anthocyanins and more. Expert Rev Gastroenterol Hepatol. 2018 Sep 26:1-18.	Review
15)	Pan P, Huang YW, Oshima K, Yearsley M, Zhang J, Yu J, Arnold M, Wang LS. (2018). Could Aspirin and Diets High in Fiber Act Synergistically to Reduce the Risk of Colon Cancer in Humans? Int J Mol Sci. 2018 Jan 6;19(1)	Review

Table 2S | List of articles excluded after screening A with specific motivations.

N°	DOCUMENTS	MOTIVATION
1)	Kubow S, Iskandar MM, Melgar-Bermudez E, Sleno L, Sabally K, Azadi B, et al. Effects of Simulated Human Gastrointestinal Digestion of Two Purple-Fleshed Potato Cultivars on Anthocyanin Composition and Cytotoxicity in Colonic Cancer and Non-Tumorigenic Cells. Nutrients. Switzerland; 2017;9.	Not performed in *in vivo* mammalian models
2)	Ombra MN, d'Acierno A, Nazzaro F, Riccardi R, Spigno P, Zaccardelli M, et al. Phenolic Composition and Antioxidant and Antiproliferative Activities of the Extracts of Twelve Common Bean (Phaseolus vulgaris L.) Endemic Ecotypes of Southern Italy before and after Cooking. Oxid Med Cell Longev. United States; 2016;2016:1398298	Not performed in *in vivo* mammalian models
3)	Xu J, Su X, Lim S, Griffin J, Carey E, Katz B, et al. Characterisation and stability of anthocyanins in purple-fleshed sweet potato P40. Food Chem. England; 2015;186:90–6.	Not performed in *in vivo* mammalian models
4)	Impei S, Gismondi A, Canuti L, Canini A. Metabolic and biological profile of autochthonous Vitis vinifera L. ecotypes. Food Funct. England; 2015;6:1526–38.	Not performed in *in vivo* mammalian models
5)	Signorelli P, Fabiani C, Brizzolari A, Paroni R, Casas J, Fabrias G, et al. Natural grape extracts regulate colon cancer cells malignancy. Nutr Cancer. United States; 2015;67:494–503.	Not performed in *in vivo* mammalian models
6)	Brown EM, Nitecki S, Pereira-Caro G, McDougall GJ, Stewart D, Rowland I, et al. Comparison of in vivo and in vitro digestion on polyphenol composition in	Using anthocyanin

	lingonberries: potential impact on colonic health. Biofactors. Netherlands; 2014;40:611–23	metabolites
7)	Correa-Betanzo J, Allen-Vercoe E, McDonald J, Schroeter K, Corredig M, Paliyath G. Stability and biological activity of wild blueberry (Vaccinium angustifolium) polyphenols during simulated in vitro gastrointestinal digestion. Food Chem. England; 2014;165:522–31.	Using anthocyanin metabolites
8)	Symonds EL, Konczak I, Fenech M. The Australian fruit Illawarra plum (Podocarpus elatus Endl., Podocarpaceae) inhibits telomerase, increases histone deacetylase activity and decreases proliferation of colon cancer cells. Br J Nutr. England; 2013;109:2117–25.	Not performed in *in vivo* mammalian models
9)	Madiwale GP, Reddivari L, Stone M, Holm DG, Vanamala J. Combined effects of storage and processing on the bioactive compounds and pro-apoptotic properties of color-fleshed potatoes in human colon cancer cells. J Agric Food Chem. United States; 2012;60:11088–96.	Not performed in *in vivo* mammalian models
10)	Hsu C-P, Shih Y-T, Lin B-R, Chiu C-F, Lin C-C. Inhibitory effect and mechanisms of an anthocyanins- and anthocyanidins-rich extract from purple-shoot tea on colorectal carcinoma cell proliferation. J Agric Food Chem. United States; 2012;60:3686–92.	Not performed in *in vivo* mammalian models
11)	Madiwale GP, Reddivari L, Holm DG, Vanamala J. Storage elevates phenolic content and antioxidant activity but suppresses antiproliferative and pro-apoptotic properties of colored-flesh potatoes against human colon cancer cell lines. J Agric Food Chem. United States; 2011;59:8155–66.	Not performed in *in vivo* mammalian models
12)	Johnson JL, Bomser JA, Scheerens JC, Giusti MM. Effect of black raspberry (Rubus occidentalis L.) extract variation conditioned by cultivar, production site, and fruit maturity stage on colon cancer cell proliferation. J Agric Food Chem. United States; 2011;59:1638–45.	Not performed in *in vivo* mammalian models
13)	Esselen M, Fritz J, Hutter M, Teller N, Baechler S, Boettler U, et al. Anthocyanin-rich extracts suppress the DNA-damaging effects of topoisomerase poisons in human colon cancer cells. Mol Nutr Food Res. Germany; 2011;55 Suppl 1:S143-53.	Not performed in *in vivo* mammalian models
14)	Cvorovic J, Tramer F, Granzotto M, Candussio L, Decorti G, Passamonti S. Oxidative stress-based cytotoxicity of delphinidin and cyanidin in colon cancer cells. Arch Biochem Biophys. United States; 2010;501:151–7.	Not performed in *in vivo* mammalian models
15)	Slavin M, Kenworthy W, Yu LL. Antioxidant properties, phytochemical composition, and antiproliferative activity of Maryland-grown soybeans with colored seed coats. J Agric Food Chem. United States; 2009;57:11174–85.	Not performed in *in vivo* mammalian models
16)	Jing P, Bomser JA, Schwartz SJ, He J, Magnuson BA, Giusti MM. Structure-function relationships of anthocyanins from various anthocyanin-rich extracts on the inhibition of colon cancer cell growth. J Agric Food Chem. United States; 2008;56:9391–8.	Not performed in *in vivo* mammalian models
17)	Cutler GJ, Nettleton JA, Ross JA, Harnack LJ, Jacobs DRJ, Scrafford CG, et al. Dietary flavonoid intake and risk of cancer in postmenopausal women: the Iowa Women's	Not using anthocyanins

	Health Study. Int J cancer. United States; 2008;123:664–71.	
18)	Dai J, Patel JD, Mumper RJ. Characterization of blackberry extract and its antiproliferative and anti-inflammatory properties. J Med Food. United States; 2007;10:258–65.	Not performed in *in vivo* mammalian models
19)	Wu QK, Koponen JM, Mykkanen HM, Torronen AR. Berry phenolic extracts modulate the expression of p21(WAF1) and Bax but not Bcl-2 in HT-29 colon cancer cells. J Agric Food Chem. United States; 2007;55:1156–63.	Not performed in *in vivo* mammalian models
20)	Yi W, Fischer J, Krewer G, Akoh CC. Phenolic compounds from blueberries can inhibit colon cancer cell proliferation and induce apoptosis. J Agric Food Chem. United States; 2005;53:7320–9.	Not performed in *in vivo* mammalian models
21)	Zhao C, Giusti MM, Malik M, Moyer MP, Magnuson BA. Effects of commercial anthocyanin-rich extracts on colonic cancer and nontumorigenic colonic cell growth. J Agric Food Chem. United States; 2004;52:6122–8.	Not performed in *in vivo* mammalian models
22)	Malik M, Zhao C, Schoene N, Guisti MM, Moyer MP, Magnuson BA. Anthocyanin-rich extract from Aronia meloncarpa E induces a cell cycle block in colon cancer but not normal colonic cells. Nutr Cancer. United States; 2003;46:186–96.	Not performed in *in vivo* mammalian models
23)	Singletary KW, Meline B. Effect of grape seed proanthocyanidins on colon aberrant crypts and breast tumors in a rat dual-organ tumor model. Nutr Cancer. United States; 2001;39:252–8.	Using proantocyanidins
24)	Seeram NP, Bourquin LD, Nair MG. Degradation products of cyanidin glycosides from tart cherries and their bioactivities. J Agric Food Chem. United States; 2001;49:4924–9.	Using anthocyanin metabolites
25)	Kamei H, Hashimoto Y, Koide T, Kojima T, Hasegawa M. Anti-tumor effect of methanol extracts from red and white wines. Cancer Biother Radiopharm. United States; 1998;13:447–52.	Not performed in *in vivo* mammalian models
26)	Briviba K, Abrahamse SL, Pool-Zobel BL, Rechkemmer G. Neurotensin-and EGF-induced metabolic activation of colon carcinoma cells is diminished by dietary flavonoid cyanidin but not by its glycosides. Nutr Cancer. United States; 2001;41:172–9.	Not performed in *in vivo* mammalian models
27)	Chatthongpisut R, Schwartz SJ, Yongsawatdigul J. Antioxidant activities and antiproliferative activity of Thai purple rice cooked by various methods on human colon cancer cells. Food Chem. England; 2015;188:99–105.	Not performed in *in vivo* mammalian models
28)	Forester SC, Choy YY, Waterhouse AL, Oteiza PI. The anthocyanin metabolites gallic acid, 3-O-methylgallic acid, and 2,4,6-trihydroxybenzaldehyde decrease human colon cancer cell viability by regulating pro-oncogenic signals. Mol Carcinog. United States; 2014;53:432–9.	Using anthocyanin metabolites
29)	Katsube N, Iwashita K, Tsushida T, Yamaki K, Kobori M. Induction of apoptosis in cancer cells by Bilberry (Vaccinium myrtillus) and the anthocyanins. J Agric Food	Not performed in *in vivo* mammalian

	Chem. United States; 2003;51:68–75.	models
30)	Lopez de Las Hazas M-C, Mosele JI, Macia A, Ludwig IA, Motilva M-J. Exploring the Colonic Metabolism of Grape and Strawberry Anthocyanins and Their in Vitro Apoptotic Effects in HT-29 Colon Cancer Cells. J Agric Food Chem. United States; 2017;65:6477–87.	Not performed in *in vivo* mammalian models
31)	Olsson ME, Gustavsson K-E, Andersson S, Nilsson A, Duan R-D. Inhibition of cancer cell proliferation in vitro by fruit and berry extracts and correlations with antioxidant levels. J Agric Food Chem. United States; 2004;52:7264–71.	Not performed in *in vivo* mammalian models
32)	Rezaei PF, Fouladdel S, Hassani S, Yousefbeyk F, Ghaffari SM, Amin G, et al. Induction of apoptosis and cell cycle arrest by pericarp polyphenol-rich extract of Baneh in human colon carcinoma HT29 cells. Food Chem Toxicol. England; 2012;50:1054–9.	Not performed in *in vivo* mammalian models
33)	Seeram NP, Adams LS, Hardy ML, Heber D. Total cranberry extract versus its phytochemical constituents: antiproliferative and synergistic effects against human tumor cell lines. J Agric Food Chem. United States; 2004;52:2512–7.	Not performed in *in vivo* mammalian models
34)	Zu X, Zhang Z, Zhang X, Yoshioka M, Yang Y, Li J. Anthocyanins extracted from Chinese blueberry (Vaccinium uliginosum L.) and its anticancer effects on DLD-1 and COLO205 cells. Chin Med J (Engl). China; 2010;123:2714–9.	Not performed in *in vivo* mammalian models
35)	Mazewski C, Liang K, Gonzalez de Mejia E. Comparison of the effect of chemical composition of anthocyanin-rich plant extracts on colon cancer cell proliferation and their potential mechanism of action using in vitro, in silico, and biochemical assays. *Food Chem* 2018, 242:378–388.	Not performed in *in vivo* mammalian models
36)	Venancio VP, Cipriano PA, Kim H, Antunes LMG, Talcott ST, Mertens-Talcott SU: Cocoplum (Chrysobalanus icaco L.) anthocyanins exert anti-inflammatory activity in human colon cancer and non-malignant colon cells. *Food Funct* 2017, 8:307–314.	Not performed in *in vivo* mammalian models
37)	Yun J-M, Afaq F, Khan N, Mukhtar H: Delphinidin, an anthocyanidin in pigmented fruits and vegetables, induces apoptosis and cell cycle arrest in human colon cancer HCT116 cells. *Mol Carcinog* 2009, 48:260–270.	Not performed in *in vivo* mammalian models
38)	Shin DY, Lee WS, Lu JN, Kang MH, Ryu CH, Kim GY, Kang HS, Shin SC, Choi YH: Induction of apoptosis in human colon cancer HCT-116 cells by anthocyanins through suppression of Akt and activation of p38-MAPK. *Int J Oncol* 2009, 35:1499–1504.	Not performed in *in vivo* mammalian models
39)	Anwar S, Fratantonio D, Ferrari D, Saija A, Cimino F, Speciale A: Berry anthocyanins reduce proliferation of human colorectal carcinoma cells by inducing caspase-3 activation and p21 upregulation. *Mol Med Rep* 2016, 14:1397–1403.	Not performed in *in vivo* mammalian models
40)	Shin DY, Lu JN, Kim G-Y, Jung JM, Kang HS, Lee WS, Choi YH: Anti-invasive activities of anthocyanins through modulation of tight junctions and suppression of matrix metalloproteinase activities in HCT-116 human colon carcinoma cells. *Oncol Rep* 2011, 25:567–572.	Not performed in *in vivo* mammalian models

41)	Renis M, Calandra L, Scifo C, Tomasello B, Cardile V, Vanella L, Bei R, La Fauci L, Galvano F: Response of cell cycle/stress-related protein expression and DNA damage upon treatment of CaCo2 cells with anthocyanins. *Br J Nutr* 2008, 100:27–35.	Not performed in *in vivo* mammalian models
42)	Jang CH, Lee IA, Ha YR, Lim J, Sung M-K, Lee S-J, Kim J-S: PGK1 induction by a hydrogen peroxide treatment is suppressed by antioxidants in human colon carcinoma cells. *Biosci Biotechnol Biochem* 2008, 72:1799–1808.	Not performed in *in vivo* mammalian models
43)	Wang L-S, Kuo C-T, Cho S-J, Seguin C, Siddiqui J, Stoner K, Weng Y-I, Huang TH-M, Tichelaar J, Yearsley M, Stoner GD, Huang Y-W: Black raspberry-derived anthocyanins demethylate tumor suppressor genes through the inhibition of DNMT1 and DNMT3B in colon cancer cells. *Nutr Cancer* 2013, 65:118–125.	Not performed in *in vivo* mammalian models
44)	Chen L, Jiang B, Zhong C, Guo J, Zhang L, Mu T, Zhang Q, Bi X: Chemoprevention of colorectal cancer by black raspberry anthocyanins involved the modulation of gut microbiota and SFRP2 demethylation. *Carcinogenesis* 2018, 39:471–481.	Not performed in *in vivo* mammalian models
45)	Lea MA, Ibeh C, desBordes C, Vizzotto M, Cisneros-Zevallos L, Byrne DH, Okie WR, Moyer MP: Inhibition of growth and induction of differentiation of colon cancer cells by peach and plum phenolic compounds. *Anticancer Res* 2008, 28:2067–2076.	Not performed in *in vivo* mammalian models
46)	Banerjee N, Kim H, Talcott S, Mertens-Talcott S: Pomegranate polyphenolics suppressed azoxymethane-induced colorectal aberrant crypt foci and inflammation: possible role of miR-126/VCAM-1 and miR-126/PI3K/AKT/mTOR. *Carcinogenesis* 2013, 34:2814–2822.	Not using ACs
	ARTICLES EXCLUDED AFTER ELIGIBILITY CHECK	
1)	Cai H, Thomasset SC, P-Berry D, Garcea G, Brown K, Steward WP, et al. Determination of anthocyanins in the urine of patients with colorectal liver metastases after administration of bilberry extract. Biomed Chromatogr. England; 2011;25:660–3.	Statistics is missing

Table 3S | List of articles excluded after screening B with specific motivations.

N°	DOCUMENTS	MOTIVATIONS
1)	Fernandez J, Garcia L, Monte J, Villar CJ, Lombo F. Functional Anthocyanin-Rich Sausages Diminish Colorectal Cancer in an Animal Model and Reduce Pro-Inflammatory Bacteria in the Intestinal Microbiota. Genes (Basel). Switzerland; 2018;9.	Not using pure AC molecules
2)	Asadi K, Ferguson LR, Philpott M, Karunasinghe N. Cancer-preventive Properties of an Anthocyanin-enriched Sweet Potato in the APC(MIN) Mouse Model. J cancer Prev. Korea (South); 2017;22:135–46.	Not using pure AC molecules<br><br>Not reporting anticancer effects by a definite biological interaction
3)	Kubow S, Iskandar MM, Melgar-Bermudez E, Sleno L, Sabally K, Azadi B, et al. Effects of Simulated Human Gastrointestinal Digestion of Two Purple-Fleshed Potato Cultivars on Anthocyanin Composition and Cytotoxicity in Colonic Cancer and Non-Tumorigenic Cells. Nutrients. Switzerland; 2017;9.	Not using pure AC molecules
4)	Lippert E, Ruemmele P, Obermeier F, Goelder S, Kunst C, Rogler G, et al. Anthocyanins Prevent Colorectal Cancer Development in a Mouse Model. Digestion. Switzerland; 2017;95:275–80.	Using food extracts or mixtures
5)	Ombra MN, d'Acierno A, Nazzaro F, Riccardi R, Spigno P, Zaccardelli M, et al. Phenolic Composition and Antioxidant and Antiproliferative Activities of the Extracts of Twelve Common Bean (Phaseolus vulgaris L.) Endemic Ecotypes of Southern Italy before and after Cooking. Oxid Med Cell Longev. United States; 2016;2016:1398298.	Using food extracts or mixtures
6)	Xu M, Chen Y-M, Huang J, Fang Y-J, Huang W-Q, Yan B, et al. Flavonoid intake from vegetables and fruits is inversely associated with colorectal cancer risk: a case-	Not using pure AC

	control study in China. Br J Nutr. England; 2016;116:1275–87.	molecules
7)	Nimptsch K, Zhang X, Cassidy A, Song M, O'Reilly EJ, Lin JH, et al. Habitual intake of flavonoid subclasses and risk of colorectal cancer in 2 large prospective cohorts. Am J Clin Nutr. United States; 2016;103:184–91.	Not reporting anticancer effects by a definite biological interaction
8)	Charepalli V, Reddivari L, Radhakrishnan S, Vadde R, Agarwal R, Vanamala JKP. Anthocyanin-containing purple-fleshed potatoes suppress colon tumorigenesis via elimination of colon cancer stem cells. J Nutr Biochem. United States; 2015;26:1641–9.	Not using pure AC molecules
9)	Xu J, Su X, Lim S, Griffin J, Carey E, Katz B, et al. Characterisation and stability of anthocyanins in purple-fleshed sweet potato P40. Food Chem. England; 2015;186:90–6.	Not performed in mammalian models<br>Not reporting anticancer effects by a definite biological interaction
10)	Impei S, Gismondi A, Canuti L, Canini A. Metabolic and biological profile of autochthonous Vitis vinifera L. ecotypes. Food Funct. England; 2015;6:1526–38.	Not using pure AC molecules
11)	Shi N, Clinton SK, Liu Z, Wang Y, Riedl KM, Schwartz SJ, et al. Strawberry phytochemicals inhibit azoxymethane/dextran sodium sulfate-induced colorectal carcinogenesis in Crj: CD-1 mice. Nutrients. Switzerland; 2015;7:1696–715.	Not using pure AC molecules
12)	Signorelli P, Fabiani C, Brizzolari A, Paroni R, Casas J, Fabrias G, et al. Natural grape extracts regulate colon cancer cells malignancy. Nutr Cancer. United States; 2015;67:494–503.	Using food extracts or mixtures
13)	Brown EM, Nitecki S, Pereira-Caro G, McDougall GJ, Stewart D, Rowland I, et al. Comparison of in vivo and in vitro digestion on polyphenol composition in lingonberries: potential impact on colonic health. Biofactors. Netherlands; 2014;40:611–23.	Using food extracts or mixtures
14)	Correa-Betanzo J, Allen-Vercoe E, McDonald J, Schroeter K, Corredig M, Paliyath G. Stability and biological activity of wild blueberry (Vaccinium angustifolium) polyphenols during simulated in vitro gastrointestinal digestion. Food Chem. England; 2014;165:522–31.	Using food extracts or mixtures
15)	Banerjee N, Kim H, Talcott S, Mertens-Talcott S. Pomegranate polyphenolics suppressed azoxymethane-induced colorectal aberrant crypt foci and inflammation: possible role of miR-126/VCAM-1 and miR-126/PI3K/AKT/mTOR. Carcinogenesis. England; 2013;34:2814–22.	Using food extracts or mixtures
16)	Symonds EL, Konczak I, Fenech M. The Australian fruit Illawarra plum (Podocarpus elatus Endl., Podocarpaceae) inhibits telomerase, increases histone deacetylase activity and decreases proliferation of colon cancer cells. Br J Nutr. England; 2013;109:2117–25.	Using food extracts or mixtures
17)	Madiwale GP, Reddivari L, Stone M, Holm DG, Vanamala J. Combined effects of storage and processing on the bioactive compounds and pro-apoptotic properties of	Using food extracts or

	color-fleshed potatoes in human colon cancer cells. J Agric Food Chem. United States; 2012;60:11088–96.	mixtures
18)	Hsu C-P, Shih Y-T, Lin B-R, Chiu C-F, Lin C-C. Inhibitory effect and mechanisms of an anthocyanins- and anthocyanidins-rich extract from purple-shoot tea on colorectal carcinoma cell proliferation. J Agric Food Chem. United States; 2012;60:3686–92.	Using food extracts or mixtures
19)	Madiwale GP, Reddivari L, Holm DG, Vanamala J. Storage elevates phenolic content and antioxidant activity but suppresses antiproliferative and pro-apoptotic properties of colored-flesh potatoes against human colon cancer cell lines. J Agric Food Chem. United States; 2011;59:8155–66.	Using food extracts or mixtures
20)	Johnson JL, Bomser JA, Scheerens JC, Giusti MM. Effect of black raspberry (Rubus occidentalis L.) extract variation conditioned by cultivar, production site, and fruit maturity stage on colon cancer cell proliferation. J Agric Food Chem. United States; 2011;59:1638–45.	Using food extracts or mixtures
21)	Esselen M, Fritz J, Hutter M, Teller N, Baechler S, Boettler U, et al. Anthocyanin-rich extracts suppress the DNA-damaging effects of topoisomerase poisons in human colon cancer cells. Mol Nutr Food Res. Germany; 2011;55 Suppl 1:S143-53.	Using food extracts or mixtures
22)	Cai H, Thomasset SC, P-Berry D, Garcea G, Brown K, Steward WP, et al. Determination of anthocyanins in the urine of patients with colorectal liver metastases after administration of bilberry extract. Biomed Chromatogr. England; 2011;25:660–3.	Using food extracts or mixtures
23)	Cvorovic J, Tramer F, Granzotto M, Candussio L, Decorti G, Passamonti S. Oxidative stress-based cytotoxicity of delphinidin and cyanidin in colon cancer cells. Arch Biochem Biophys. United States; 2010;501:151–7.	Not reporting anticancer effects by a definite biological interaction
24)	Slavin M, Kenworthy W, Yu LL. Antioxidant properties, phytochemical composition, and antiproliferative activity of Maryland-grown soybeans with colored seed coats. J Agric Food Chem. United States; 2009;57:11174–85.	Using food extracts or mixtures
25)	Thomasset S, Berry DP, Cai H, West K, Marczylo TH, Marsden D, et al. Pilot study of oral anthocyanins for colorectal cancer chemoprevention. Cancer Prev Res (Phila). United States; 2009;2:625–33.	Using food extracts or mixtures
26)	Jing P, Bomser JA, Schwartz SJ, He J, Magnuson BA, Giusti MM. Structure-function relationships of anthocyanins from various anthocyanin-rich extracts on the inhibition of colon cancer cell growth. J Agric Food Chem. United States; 2008;56:9391–8.	Using food extracts or mixtures
27)	Cutler GJ, Nettleton JA, Ross JA, Harnack LJ, Jacobs DRJ, Scrafford CG, et al. Dietary flavonoid intake and risk of cancer in postmenopausal women: the Iowa Women's Health Study. Int J cancer. United States; 2008;123:664–71.	Using food extracts or mixtures
28)	Mursu J, Nurmi T, Tuomainen T-P, Salonen JT, Pukkala E, Voutilainen S. Intake of flavonoids and risk of cancer in Finnish men: The Kuopio Ischaemic Heart Disease Risk Factor Study. Int J cancer. United States; 2008;123:660–3.	Using food extracts or mixtures
29)	Dai J, Patel JD, Mumper RJ. Characterization of blackberry extract and its	Using food extracts or

	antiproliferative and anti-inflammatory properties. J Med Food. United States; 2007;10:258–65.	mixtures
30)	Wu QK, Koponen JM, Mykkanen HM, Torronen AR. Berry phenolic extracts modulate the expression of p21(WAF1) and Bax but not Bcl-2 in HT-29 colon cancer cells. J Agric Food Chem. United States; 2007;55:1156–63.	Using food extracts or mixtures
31)	Bobe G, Wang B, Seeram NP, Nair MG, Bourquin LD. Dietary anthocyanin-rich tart cherry extract inhibits intestinal tumorigenesis in APC(Min) mice fed suboptimal levels of sulindac. J Agric Food Chem. United States; 2006;54:9322–8.	Not reporting anticancer effects by a definite biological interaction
32)	Lala G, Malik M, Zhao C, He J, Kwon Y, Giusti MM, et al. Anthocyanin-rich extracts inhibit multiple biomarkers of colon cancer in rats. Nutr Cancer. United States; 2006;54:84–93.	Using food extracts or mixtures
33)	Yi W, Fischer J, Krewer G, Akoh CC. Phenolic compounds from blueberries can inhibit colon cancer cell proliferation and induce apoptosis. J Agric Food Chem. United States; 2005;53:7320–9.	Not reporting anticancer effects by a definite biological interaction
34)	Zhao C, Giusti MM, Malik M, Moyer MP, Magnuson BA. Effects of commercial anthocyanin-rich extracts on colonic cancer and nontumorigenic colonic cell growth. J Agric Food Chem. United States; 2004;52:6122–8.	Using food extracts or mixtures
35)	Malik M, Zhao C, Schoene N, Guisti MM, Moyer MP, Magnuson BA. Anthocyanin-rich extract from Aronia meloncarpa E induces a cell cycle block in colon cancer but not normal colonic cells. Nutr Cancer. United States; 2003;46:186–96.	Using food extracts or mixtures
36)	Kang S-Y, Seeram NP, Nair MG, Bourquin LD. Tart cherry anthocyanins inhibit tumor development in Apc(Min) mice and reduce proliferation of human colon cancer cells. Cancer Lett. Ireland; 2003;194:13–9.	Not reporting anticancer effects by a definite biological interaction
37)	Harris GK, Gupta A, Nines RG, Kresty LA, Habib SG, Frankel WL, et al. Effects of lyophilized black raspberries on azoxymethane-induced colon cancer and 8-hydroxy-2'-deoxyguanosine levels in the Fischer 344 rat. Nutr Cancer. United States; 2001;40:125–33.	Not using pure AC molecules
38)	Hagiwara A, Yoshino H, Ichihara T, Kawabe M, Tamano S, Aoki H, et al. Prevention by natural food anthocyanins, purple sweet potato color and red cabbage color, of 2-amino-1-methyl-6-phenylimidazo[4,5-b]pyridine (PhIP)-associated colorectal carcinogenesis in rats initiated with 1,2-dimethylhydrazine. J Toxicol Sci. Japan; 2002;27:57–68.	Using food extracts or mixtures
39)	Singletary KW, Meline B. Effect of grape seed proanthocyanidins on colon aberrant crypts and breast tumors in a rat dual-organ tumor model. Nutr Cancer. United States; 2001;39:252–8.	Not using pure AC molecules
40)	Seeram NP, Bourquin LD, Nair MG. Degradation products of cyanidin glycosides from tart cherries and their bioactivities. J Agric Food Chem. United States; 2001;49:4924–	Not reporting anticancer effects by a definite biological

	9.	interaction
41)	Hagiwara A, Miyashita K, Nakanishi T, Sano M, Tamano S, Kadota T, et al. Pronounced inhibition by a natural anthocyanin, purple corn color, of 2-amino-1-methyl-6-phenylimidazo[4,5-b]pyridine (PhIP)-associated colorectal carcinogenesis in male F344 rats pretreated with 1,2-dimethylhydrazine. Cancer Lett. Ireland; 2001;171:17–25.	Not reporting anticancer effects by a definite biological interaction
42)	Kamei H, Hashimoto Y, Koide T, Kojima T, Hasegawa M. Anti-tumor effect of methanol extracts from red and white wines. Cancer Biother Radiopharm. United States; 1998;13:447–52.	Using food extracts or mixtures
43)	Lea MA, Ibeh C, desBordes C, Vizzotto M, Cisneros-Zevallos L, Byrne DH, Okie WR, Moyer MP: Inhibition of growth and induction of differentiation of colon cancer cells by peach and plum phenolic compounds. *Anticancer Res* 2008, 28:2067–2076.	Not reporting anticancer effects by a definite biological interaction
44)	Chatthongpisut R, Schwartz SJ, Yongsawatdigul J. Antioxidant activities and antiproliferative activity of Thai purple rice cooked by various methods on human colon cancer cells. Food Chem. England; 2015;188:99–105.	Using food extracts;
45)	Cooke D, Schwarz M, Boocock D, Winterhalter P, Steward WP, Gescher AJ, et al. Effect of cyanidin-3-glucoside and an anthocyanin mixture from bilberry on adenoma development in the ApcMin mouse model of intestinal carcinogenesis--relationship with tissue anthocyanin levels. Int J cancer. United States; 2006;119:2213–20.	Not reporting experimental data about anticancer effect by a definite biological interaction
46)	Forester SC, Choy YY, Waterhouse AL, Oteiza PI. The anthocyanin metabolites gallic acid, 3-O-methylgallic acid, and 2,4,6-trihydroxybenzaldehyde decrease human colon cancer cell viability by regulating pro-oncogenic signals. Mol Carcinog. United States; 2014;53:432–9.	Using AC metabolites
47)	Katsube N, Iwashita K, Tsushida T, Yamaki K, Kobori M. Induction of apoptosis in cancer cells by Bilberry (Vaccinium myrtillus) and the anthocyanins. J Agric Food Chem. United States; 2003;51:68–75.	Not reporting experimental data about anticancer effect by a definite biological interaction;
48)	Lim S, Xu J, Kim J, Chen T-Y, Su X, Standard J, et al. Role of anthocyanin-enriched purple-fleshed sweet potato p40 in colorectal cancer prevention. Mol Nutr Food Res. Germany; 2013;57:1908–17.	Not pure ACs
49)	Lopez de Las Hazas M-C, Mosele JI, Macia A, Ludwig IA, Motilva M-J. Exploring the Colonic Metabolism of Grape and Strawberry Anthocyanins and Their in Vitro Apoptotic Effects in HT-29 Colon Cancer Cells. J Agric Food Chem. United States; 2017;65:6477–87.	Food extracts;<br><br>AC methabolites
50)	Olsson ME, Gustavsson K-E, Andersson S, Nilsson A, Duan R-D. Inhibition of cancer cell proliferation in vitro by fruit and berry extracts and correlations with antioxidant	Not reporting experimental data about anticancer effect

	levels. J Agric Food Chem. United States; 2004;52:7264–71.	by a definite biological interaction;
51)	Rezaei PF, Fouladdel S, Hassani S, Yousefbeyk F, Ghaffari SM, Amin G, et al. Induction of apoptosis and cell cycle arrest by pericarp polyphenol-rich extract of Baneh in human colon carcinoma HT29 cells. Food Chem Toxicol. England; 2012;50:1054–9.	Using food extracts;
52)	Seeram NP, Adams LS, Hardy ML, Heber D. Total cranberry extract versus its phytochemical constituents: antiproliferative and synergistic effects against human tumor cell lines. J Agric Food Chem. United States; 2004;52:2512–7.	Not reporting experimental data about anticancer effect by a definite biological interaction;
53)	Zu X, Zhang Z, Zhang X, Yoshioka M, Yang Y, Li J. Anthocyanins extracted from Chinese blueberry (Vaccinium uliginosum L.) and its anticancer effects on DLD-1 and COLO205 cells. Chin Med J (Engl). China; 2010;123:2714–9.	Not using pure AC molecules
54)	Chen L, Jiang B, Zhong C, Guo J, Zhang L, Mu T, Zhang Q, Bi X: Chemoprevention of colorectal cancer by black raspberry anthocyanins involved the modulation of gut microbiota and SFRP2 demethylation. *Carcinogenesis* 2018, 39:471–481	Not using pure AC molecules
55)	Wang L-S, Kuo C-T, Cho S-J, Seguin C, Siddiqui J, Stoner K, Weng Y-I, Huang TH-M, Tichelaar J, Yearsley M, Stoner GD, Huang Y-W: Black raspberry-derived anthocyanins demethylate tumor suppressor genes through the inhibition of DNMT1 and DNMT3B in colon cancer cells. *Nutr Cancer* 2013, 65:118–125.	Not using pure AC molecules
56)	Qin XX, Zhang MY, Han YY, Hao JH, Liu CJ, Fan SX. (2018). Beneficial Phytochemicals with Anti-Tumor Potential Revealed through Metabolic Profiling of New Red Pigmented Lettuces (Lactuca sativa L). Int J Mol Sci. 2018 Apr 11;19(4)	Not using pure AC molecules
57)	Grimes KL, Stuart CM, McCarthy JJ, Kaur B, Cantu EJ, Forester SC. Enhancing the Cancer Cell Growth Inhibitory Effects of Table Grape Anthocyanins. J Food Sci. 2018 Sep;83(9):2369-2374. doi: 10.1111/1750-3841.14294. Epub 2018 Aug 2.	Not reporting experimental data about anticancer effect by a definite biological interaction
